# *Paeonia peregrina* Mill Petals as a New Source of Biologically Active Compounds: Chemical Characterization and Skin Regeneration Effects of the Extracts

**DOI:** 10.3390/ijms241411764

**Published:** 2023-07-21

**Authors:** Tatjana Marković, Natalija Čutović, Tamara Carević, Uroš Gašić, Dejan Stojković, Jingqi Xue, Aleksandra Jovanović

**Affiliations:** 1 Institute for Medicinal Plants Research “Dr Josif Pančić”, Tadeuša Košćuška 1, 11000 Belgrade, Serbia; ncutovic@mocbilja.rs; 2 Department of Plant Physiology, Institute for Biological Research “Siniša Stanković”—National Institute of Republic of Serbia, University of Belgrade, Bulevar Despota Stefana 142, 11000 Belgrade, Serbia; tamara.carevic@ibiss.bg.ac.rs (T.C.); uros.gasic@ibiss.bg.ac.rs (U.G.); dejanbio@ibiss.bg.ac.rs (D.S.); 3 Key Laboratory of Biology and Genetic Improvement of Horticultural Crops, Institute of Vegetables and Flowers, Chinese Academy of Agricultural Sciences, Ministry Agriculture and Rural Affairs, Beijing 100081, China; xuejingqi@caas.cn; 4 Institute for the Application of Nuclear Energy INEP, University of Belgrade, Banatska 31b, Zemun, 11080 Belgrade, Serbia; ajovanovic@inep.co.rs

**Keywords:** petals, herbaceous peony, biological activities, chemical composition

## Abstract

*Paeonia peregrina* Mill. is a perennial herbaceous plant species, known for the medicinal value of all of its plant parts, although the chemical composition of the petals is unknown. This study aimed to determine the chemical fingerprint of the petals and also establish the optimal extraction parameters, extraction medium, and extraction method for petals collected from different localities in Serbia. The optimization was performed in order to acquire extracts that are rich in the contents of total polyphenol content (TPC) and total flavonoid content (TFC), and also exhibit strong antioxidant activity. In addition, the influence of the extracts on several human skin pathogens was evaluated, as well as their ability to aid wound closure and act as anti-inflammatory agents. Both the extraction medium and the applied technique significantly influenced the skin-beneficial biological activities, while methanol proved to be a more favorable extraction medium. In conclusion, the extraction conditions that yielded the extract with the richest phenolic content with satisfactory biological potential varied between the assays, while the most promising locality in Serbia for the collection of *P. peregrina* petals was Pančevo (South Banat).

## 1. Introduction

The only genus in the family *Paeoniaceae* is *Paeonia* L. The most recent classification suggested its division into the subgenus *Moutan,* comprising 9 woody peony species, and the subgenus *Paeonia,* comprising 25 herbaceous peony species [1]. According to the Plant List Database [2], members of this genus are primarily encountered in temperate Asia, southern Europe, and western North America.

In Serbia, only herbaceous peonies spontaneously grow. Following the last assessments of the state and number of their populations, relevant threats, and other factors associated with their protection, the following five taxa were confirmed: *Paeonia peregrina* Mill, *Paeonia tenuifolia* L., *Paeonia officinalis* L. subsp. *officinalis*, *Paeonia officinalis* subsp. *banatica* (Rochel) Soó, and *Paeonia mascula* (L.) Mill [3]. All of them are listed in the Red Book [4], meaning that their collection from nature is strictly protected by law [5] and requires permission from the Ministry of Environmental Protection of the Republic of Serbia.

**Figure 1:**
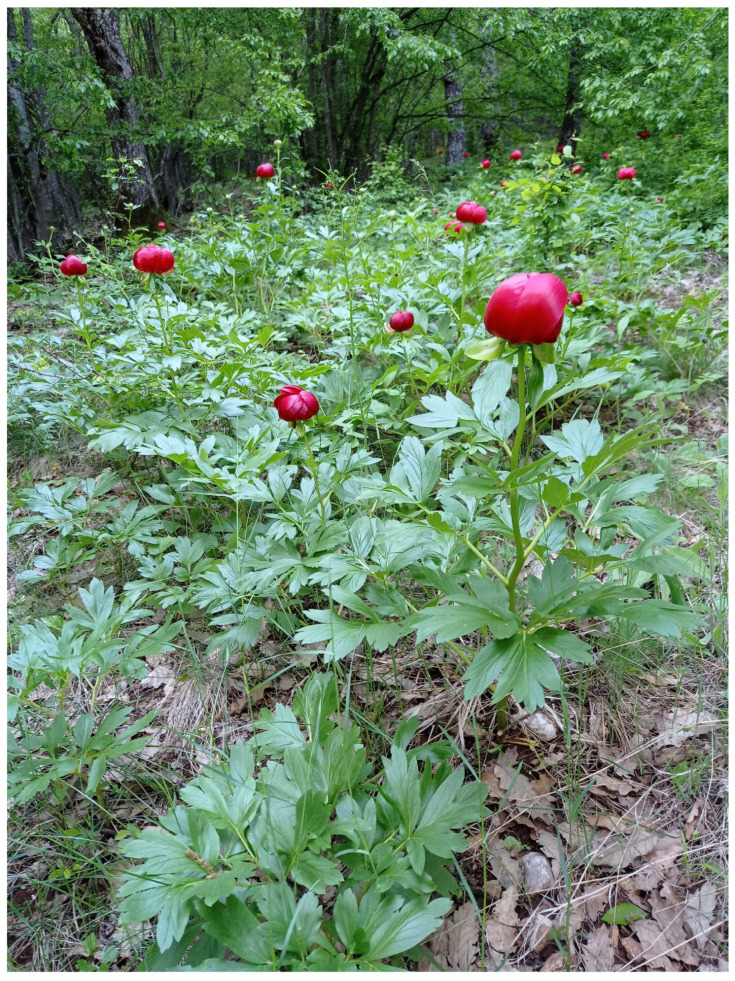
Flowering plants of the Paeonia peregrina Mill population in the locality of Krivi vir, Serbia (May, 2022).

*P. peregrina* Mill., also known as the Balkan or Kosovo peony, is considered the most widespread in Serbia. The eastern part of the country (Krivi vir, Skrobnica, Golina, and Pirot) and Kosovo host the species’ densest populations. The 30–70 cm tall stems of this herbaceous species are characterized by solitary flowers with cup-shaped corollas made up of 7–10 red or dark red petals [6]. The flower typically blooms in May for 7–15 days, which is a relatively short period of time (Figure 1).

As plants of the genus *Paeonia* have become more popular for their medicinal and edible properties, in recent decades, extensive phytochemical studies and pharmacological activity analyses have been conducted, focusing primarily on their roots and confirming a number of beneficial properties, such as the plants’ antioxidant, anti-inflammatory, antitumor, hepatoprotective, cardioprotective, and immune-modulatory qualities [7]. Leaves and stems, as well as seeds and flowers, of many members of this genus have also been studied for their chemical composition [8,9]. The seeds have attracted more attention for their edible use [8,10,11] and the flowers for both their edible [12] and ornamental uses [13].

The edible flowers have also been used for their remedial purposes for thousands of years [14]. Apart from water, which makes up 70–95% of their composition [15,16], the remaining part of the flower (i.e., the dry matter) contains variable amounts of carbohydrates, proteins, lipids, vitamins, and minerals, as well as low-molecular-weight phytochemicals [15,16]. The flowers can be used whole or for specific parts, such as the stigma and upper portion of the style or the petals. The petals are thought to be rich in vitamins and minerals, and they are also an excellent source of valuable antioxidants [17]. Notable antioxidant properties of plant extracts are mainly associated with their content of polyphenols, tocopherols, carotenoids, and ascorbic acid, but also with some macromolecules (polysaccharides and peptides) and essential oil constituents [18,19,20]. Plant-derived antioxidants might be particularly effective at preventing and repairing skin damage caused by free radicals, as they not only scavenge them but also support the skin’s defense and regenerative mechanisms [21,22]. Because of their antibacterial and antibiofilm properties, they can also prevent the spread of bacteria that are already present on the skin’s surface [21]. Those that are rich in polyphenols are also thought to have outstanding wound-healing qualities [23], particularly if they are rich in phenols, which possess astringent, antibacterial, and free-radical-scavenging properties [24]. The capacity of extracts to encourage the development and migration of healthy cells into a wound can be estimated by the wound-healing test. If a plant extract is to be applied to human skin, in addition to its anti-inflammatory effects (i.e., its impact on skin cell proliferation), it should also be tested for a cell-damaging (cytotoxic) effect, and whether it affects how specific pathogens adhere to and invade cells (to check the degree to which its binding and penetration into deeper skin cell layers could be prevented in areas treated with the extract).

Recent studies on herbaceous peonies found polyphenols to be major constituents of the petals [25,26]. The extraction of polyphenols from plant material can be influenced by a variety of factors, including the particle size and plant material characteristics, the extraction medium, the solvent-to-solid ratio, the pH value, the extraction time, the temperature, the pressure, the technique, etc. [27]. Polyphenols come in a wide variety of structural and physicochemical forms, making it impossible to establish a single extraction protocol that would work for all plant matrices. Therefore, extraction conditions should be carefully studied to maximize their yield [28].

Keeping in mind all the above factors, the aim of this study was to reveal the chemical fingerprint of the petals of wild-growing *Paeonia peregrina* Mill from various localities in Serbia and to estimate their beneficial properties for human skin.

## 2. Results and Discussion

*P. peregrina* petal extracts from different natural stands in Serbia were chemically characterized and evaluated for several skin-related properties. The outcomes are presented and discussed below.

### 2.1. Total Polyphenol Content of the Extracts

#### 2.1.1. Preliminary Screening of Factor Levels

**Figure 2:**
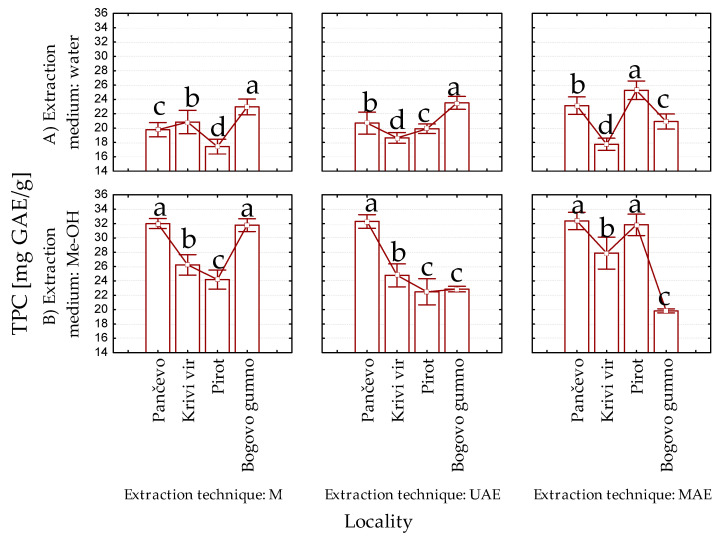
Total polyphenol content (TPC) of (**A**) water and (**B**) methanol extracts of the petals of *Paeonia peregrina* Mill.; GAE, gallic acid equivalents; M, maceration, UAE, ultrasound-assisted extraction; MAE, microwave-assisted extraction; Me-OH, methanol; values with the same letter showed no statistically significant difference (analysis of variance followed by Duncan’s post hoc test, *p* > 0.05; n = 3).

**Figure 3:**
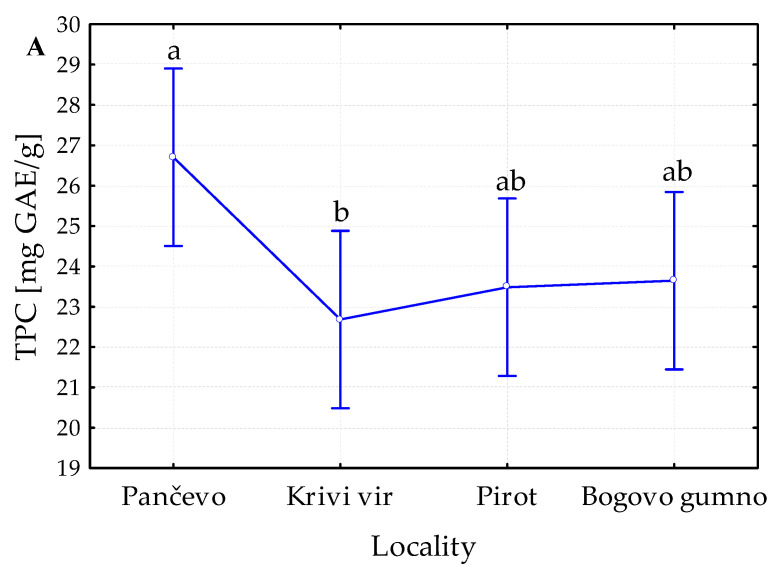

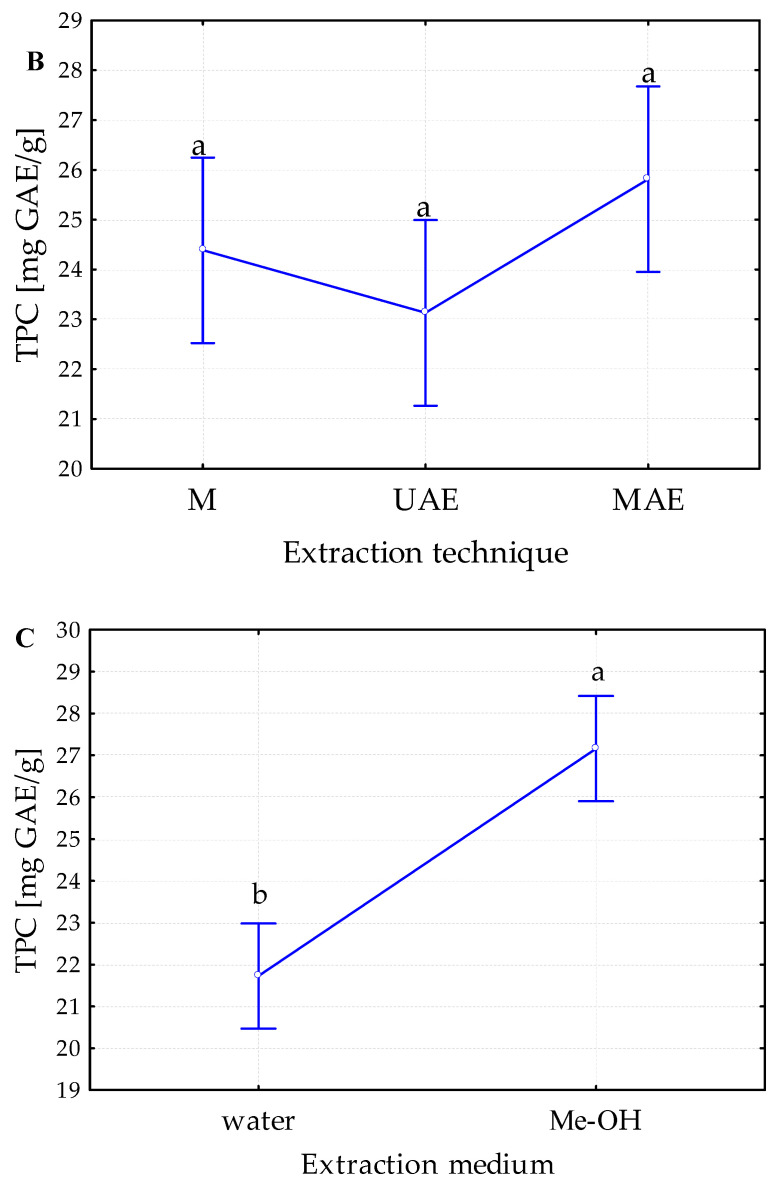
Preliminary screening of the influence of all levels of each factor, (**A**) four localities, (**B**) three extraction techniques, and (**C**) two extraction mediums, on the total polyphenol content (TPC) in petal extracts of *Paeonia peregrina* Mill.; GAE, gallic acid equivalents; M, maceration, UAE, ultrasound-assisted extraction; MAE, microwave-assisted extraction; Me-OH, methanol; values with the same letter showed no statistically significant difference (analysis of variance followed by Duncan’s post hoc test, *p* > 0.05; n = 3).

The selection of the levels of each factor (the locality, extraction medium type, and extraction technique) that gave the highest TPC values was performed. We selected two levels of each factor that provided the highest polyphenol concentration; these were subjected to further experimental design (2^3^ full factorial design). The TPC values of all the prepared extracts and the impact of the origin of the *P. peregrina* petals, the extraction method, and the extraction medium on the TPC values are presented in Figure 2 and Figure 3, respectively.

The highest TPCs for the maceration technique had water and methanol extracts from Bogovo gumno and methanol extract from Pančevo; the UAE technique had water extract from Bogovo gumno and methanol extract from Pančevo, and the MAE technique had both water and methanol extracts from Pirot and methanol extracts from Pančevo (Figure 2).

The results of the influence of locality on the TPC showed significant differences (Figure 3A). The highest TPC was recorded in the extracts from Pančevo, followed by Bogovo gumno and Pirot, with no difference between them. Therefore, these three levels of factor were primarily selected for further factorial analysis. However, the number of petals from Bogovo gumno was insufficient for further analyses, and this group was excluded from further experimental study.

The results for the influence of the extraction technique on the TPC revealed an absence of differences (Figure 3B). In addition to the first criterion (the highest TPC value), other important criteria for selecting the factor levels, such as simplicity, operational costs, and the tendency to produce free radicals, had to be included. As maceration is a simple, low-cost technique, while MAE is a fast technique without the tendency to produce free radicals, both techniques were also included in the further experimental design (2^3^, two levels of three factors).

Different polarity extraction mediums (water and methanol) were found to have a significant effect on the TPC (Figure 3C), with methanol extracts being higher in TPC than their water counterparts. Nevertheless, since certain water extracts also demonstrated some biological activities (antioxidant, antimicrobial, and antibiofilm), both types of extracts were included in the further experimental design.

#### 2.1.2. Factorial Design

**Figure 4:**
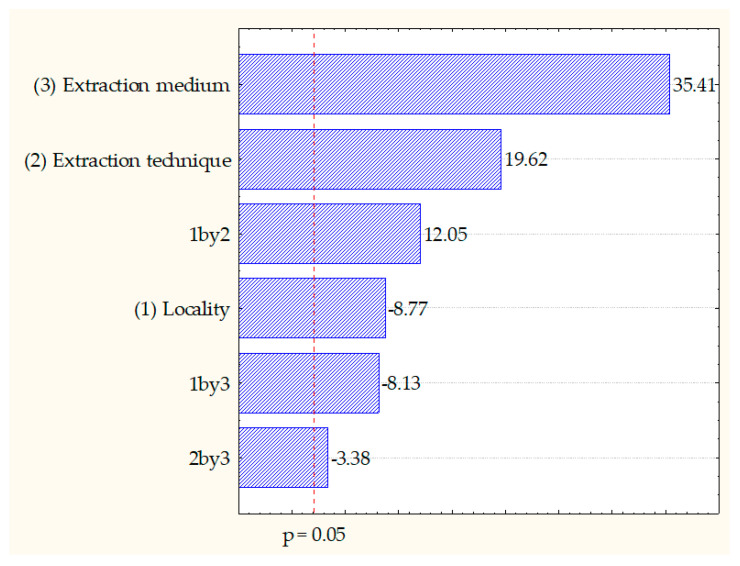
Pareto charts of selected factors and their interactions’ influence (three factors on two levels) on the total polyphenol content (TPC) in petal extracts of *Paeonia peregrina* Mill.

The influence of three factors with two levels on total polyphenols was observed through the absolute values of standardized estimated effects and presented on Pareto charts with the level of significance set at *p* < 0.05 for the factorial design (Figure 4).

The effects and corresponding regression coefficients of factors and factor interactions are listed in Table 1. Additionally, observed and predicted values for the dependent variable (TPC) are presented in Table 2.

As shown by Figure 4 and Table 1, the extraction mediums (water and methanol, variable number 3) had the most significant impact on the dependent variable (TPC), followed by extraction technique (maceration and MAE, variable number 2) and the interaction between the locality and the extraction technique (1 by 2). Locality (as single variable number 1) and the interactions between the locality and the extraction medium type (1 by 3) and between the extraction technique and the extraction medium type (2 by 3) had a lower influence on the total polyphenol content.

The obtained results are consistent with data from preexisting studies, which show that the type of extraction medium (methanol, water) affects the TPC in the tested herbaceous plant extracts of *Urtica dioica* [28]. In addition, methanol has already shown higher extraction efficacy for polyphenols compared to a polar extraction medium, such as water [29]; it promoted the solubility of polyphenols, thus increasing the efficiency of their extraction [30]. Similarly to our findings, in studies with the petals of *Rosa rugosa* [31] and *Hibiscus sabdariffa* [32], the methanol extracts had higher TPC values compared to the water extracts. Additionally, during the optimization of the extraction procedure, the temperature was shown to be the dominant factor in maximizing the TPC values in other medicinal plant species [33], which is consistent with our findings. A lower maceration temperature (25 °C) compared to the MAE temperature (60 °C) resulted in lower TPC values in *P. peregrina* extracts, indicating the significant influence of the extraction technique. These findings are consistent with those from a study of polyphenol extraction from *Thymus serpyllum*, in which the extraction techniques also had a significant impact on the TPC, with novel technologies outperforming maceration [27]. Additionally, the chemical content and composition of the harvested plant material are affected by the geographic location, altitude, climate, soil, seasonal variations, cultivar, and performed agronomic practices, which can explain the significance of the plant material’s origin for the TPC of extracts [25]. The significant impact of the interaction between factors indicates that the effect of one factor was not the same at all levels of another factor. Specifically, the impact of the plant material’s origin differed when different extraction methods or extraction mediums were used, and the effect of the extraction medium also depended on the extraction technique used. Thus, it is possible to conclude that every type of plant material and extraction medium require the investigation of the appropriate extraction technique that will yield the highest TPC of the extracts.

According to the results of the 2^3^ full factorial design (the observed and predicted values of TPC in *P. peregrina* extracts are listed in Table 2), the highest observed TPC was reached under the following conditions: Pančevo (locality), MAE (extraction technique), and methanol (extraction medium), with a value of 32.35 ± 0.48 mg GAE/g. The model predicted the maximal TPC value under the same parameters (Pančevo, MAE, and methanol) to be 32.69 mg GAE/g. Since the differences between the predicted and measured values (Table 2) were all less than 2%, a full factorial design may be recommended as an appropriate model for optimizing polyphenol extraction from the petals of this plant.

### 2.2. Total Flavonoid Content of the Extracts

**Figure 5:**
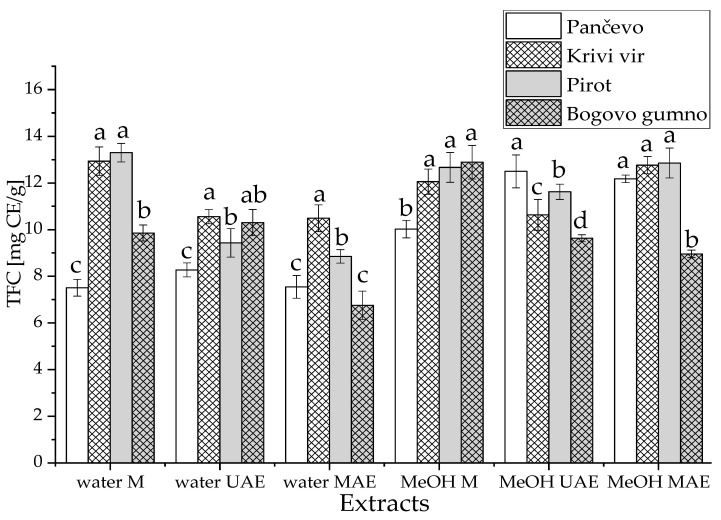
Total flavonoid content (TFC) of *Paeonia peregrina* Mill. petals; maceration (M), ultrasound-assisted extraction (UAE), and microwave-assisted extraction (MAE); values with the same letter in each group (the same extraction medium and technique) showed no statistically significant difference between different regions (*p* > 0.05; n = 3, one-way ANOVA, analysis of variance, Duncan’s post hoc test).

The effects of the origin of the *P. peregrina* petals (Pančevo, Krivi vir, Bogovo gumno, and Pirot), the extraction techniques (maceration, UAE, and MAE), and the extraction mediums (water and methanol) on the total flavonoid content (TFC) were also investigated. The TFC results for all extracts are displayed in Figure 5.

Following maceration, the petals from Krivi vir and Pirot were found to have the highest TFC for the water extracts, while the petals from Krivi vir, Pirot, and Bogovo gumno were found to have the highest TFC for the methanol extracts. In the UAE, the highest TFC was obtained in the water extract from Krivi vir and the methanol extract from Pančevo; meanwhile, for MAE, the highest TFC was observed in the water extract from Krivi vir and the methanol extracts from Pančevo, Krivi vir, and Pirot.

The origin of the plant material has been shown to have a significant impact on the TFC (Supplementary Figure S2A); the highest values were measured in the samples prepared using petals from Krivi vir and Pirot. The extraction procedures had a significant influence on the flavonoid content, which was higher after maceration compared to UAE and MAE, which did not differ (Supplementary Figure S2B). This finding is consistent with previous studies, in which high temperatures negatively influenced the TFC [34] and flavonoids were sensitive to temperature [35]. These findings support the use of maceration as an extraction technique for *P. peregrina* petals, particularly for flavonoid-rich extracts. Additionally, a higher TFC was achieved in *P. peregrina* petals prepared using methanol as an extraction medium (Supplementary Figure S2C), in the same manner as in the case of TPC. Similarly, the content of flavonoids was significantly higher in the methanol extracts of the petals of *Crocus sativus* compared to their water counterparts [36]. Alcohols, in particular, react with flavonoid compounds through non-covalent interactions, increasing their rapid diffusion into the extraction surroundings [37,38]. Because of their capacity to induce a larger flavonoid release, alcohols are the most commonly utilized extraction mediums for flavonoids [39]. However, the extraction efficiency is also affected by the type of flavonoid compound to be recovered, with methanol being used to extract polar flavonoids such as flavonoid glycosides and aglycones [40].

### 2.3. Chemical Composition

To the best of our knowledge, this is the first study of the chemical composition of the petals of wild-growing *P. peregrina* from different localities in Serbia. Therefore, the results are presented in detail. A total of 102 compounds were found in the methanol extracts of the petals of *P. peregrina* from Pančevo, which showed the highest TPC value (Table 3). They were divided into the following major groups: phenolic acids (compounds **1**–**46**), flavonoid glycosides and aglycones (compounds **47**–**68**), anthocyanins and anthocyanidins (compounds **69**–**86**), terpene derivatives (compounds **87**–**95**), and other compounds (compounds **96**–**102**). The chromatograms in negative and positive ionization modes are shown in Supplementary Figure S3.

**Phenolic acids**. With 46 known chemical compounds, this was the most abundant group in the studied extracts. Chromatographic traits that matched the standards for gallic acid, ellagic acid, and *p*-coumaric acid were used for the successful identification of compounds **3**, **26**, and **40**, respectively. Compounds **3**, **12**, **15**, **16**, **30**, **33**, **37**, **38**, **39**, and **42** have previously been reported to be constituents of many herbaceous peony species [41,42], but the compounds **6**, **7**, **14**, **17**, **20**, **23**, **24**, **28**, **41**, **43**, **44**, and **46** are now tentatively recognized for the first time in the petals of *P. peregrina*. Another two compounds, **26** and **40**, were also recently found in the petals of the herbaceous peony species *P. tenuifolia* [25]. Using a molecular ion at 331 *m/z*, compounds **1** and **2** (galloyl-hexoside) were detected at 0.57 and 0.87 min, respectively. These two compounds provided MS^2^ base peaks at 169 *m/z*, created by the loss of 162 Da (hexosyl group). MS^3^ base peaks were found at 125 *m/z* (loss of CO_2_—44 Da). Digallic acid derivatives were discovered as compounds **5** (3.00 min), **11** (3.49 min), **25** (4.64 min), **35** (5.25 min), and **45** (6.49 min), and their fragmentations were recently validated [43,44]. Compounds **9** (3.34 min), **31** (4.89 min), and **36** (5.25 min) all had the same exact masses (183 *m/z*) and similar fragmentation patterns, indicating that they are isomers of methyl gallate. The MS^2^ base peak, which can either be gallic acid residue or methylene residue, was discovered at 168 *m/z* in all three cases. Three other compounds, 13 (3.62 min), 18 (4.02 min), and 22 (4.34 min), were identified as being the isomers of trigalloyl-hexoside based on their masses (635 *m/z*) and fragmentation patterns. Each of them had an MS2 base peak at 465 *m/z* so could be trigallic acid or hexose residues. As pentagalloyl-hexoside isomers, compounds **29** (4.78 min), **32** (4.89 min), and **34** (5.19 min) had the same precise masses (183 *m/z*) and exhibited comparable fragmentation patterns. The MS^3^ base peak at 617 *m/z* was found for all of them, and the pentagallic acid or hexoside residue is most likely responsible for that peak value. The isomers of digalloyl-hexoside (**4**, **10**), galloyl-norbigenin (**8**, **19**), and tetragalloyl-hexoside (**21**, **27**), with respective MS2 peaks of 169, 153, and 617 *m/z*, respectively, were also discovered. The remaining 10 phenolic acids (**14**, **17**, **18**, **20**, **22**, **27**, **34**, **35**, and **39**) were identified based on their typical MS spectra and fragmentation patterns.

**Flavonoid glycosides and aglycones**. This was the chemical group with the second-highest number of compounds in the studied extract, with a total of 22 compounds. Most of them were already confirmed in peonies but in other plant parts: compound **49** in the roots of *P. lactiflora* [45], **55** and **58** in the roots and aerial parts of *Paeonia kesrounansis*, *Paeonia arientina* [46], and *Paeonia parnassica* [47], and **50** in the seeds of *P. lactiflora* [38]. Compound **60** was recently discovered by our group in the petals of *Paeonia tenuifolia* [25], while compounds **53**, **62**, **63**, **64**, **67**, and **68** were previously confirmed in the petals of this plant species [39,40,41,42]. The remaining compounds assigned to this group have never previously been connected to herbaceous peonies, particularly the petals.

**Anthocyanins and anthocyanidins**. In *Paeonia* taxa, 11 out of the 18 compounds from this group had already been verified. The following six have previously been used to distinguish herbaceous peonies; compounds **69**, **70**, and **75** are confirmed as constituents of the flowers [48] and **79** of the roots of *P. lactiflora* [49], while compounds **81** and **86** are confirmed as constituents of the petals of *P. tenuifolia* [25]. Recently, another three compounds (**71**, **77**, and **85**) were identified in the petals of *P. tenuifolia* [25]. Compound **76** was confirmed in flowers of the woody species *Paeonia suffruticosa* [49], while **84** was found in a cultivar of the same species [50].

**Terpene derivatives**. This group contained 11 compounds, all of which have been previously identified as typical for the *Paeonia* taxonomy. Compounds **88** and **89** were reported as constituents of *P. lactiflora* roots and seeds, respectively [51]. Compounds **87** and **94** were also detected in roots of *P. lactiflora* [44] and *Paeonia daurica* [52], respectively. The only compounds found in the petals of herbaceous peonies were **90**, **91**, **92**, **93**, and **95**, and they were discovered recently by our group, in the petals of *P. tenuifolia* [25].

**Other compounds**. The following seven chemicals were tentatively identified for the first time in the petals of *P. peregrina*: citric acid (**96**), shikimic acid (**97**), apiopaeonoside (**98**), paeonoside (**99**), paeonol (**100**), picrocrocinic acid (**101**), and (+)-paeonilactone B (**102**). Compounds **96**, **99**, and **101** were previously found in the roots [42,51,53], while (+)-Paeonilactone B (**102**) was found in the seed kernels of *P. lactiflora* [51]. According to a thorough review of the literature, compounds **98** (apiopaeonoside) and **100** (paeonol) were never before reported in herbaceous peonies, particularly the petals, whereas compound **97** was recently detected in the petals of *P. tenuifolia* [25].

### 2.4. Antioxidant Activity of the Extracts

**Figure 6:**
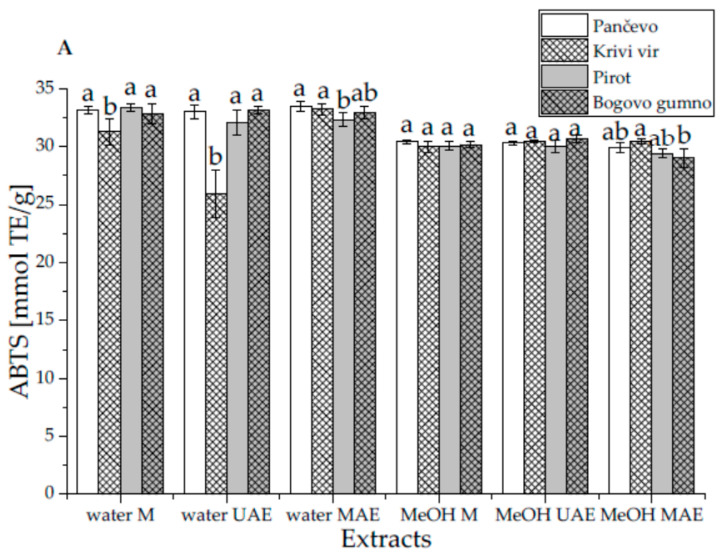

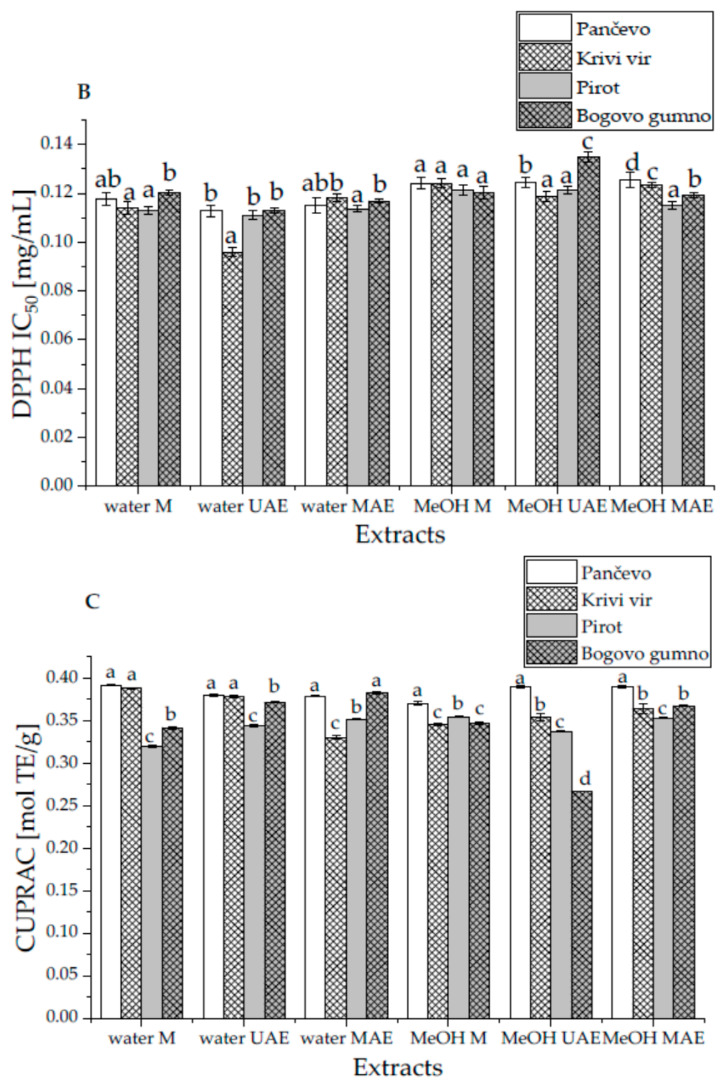

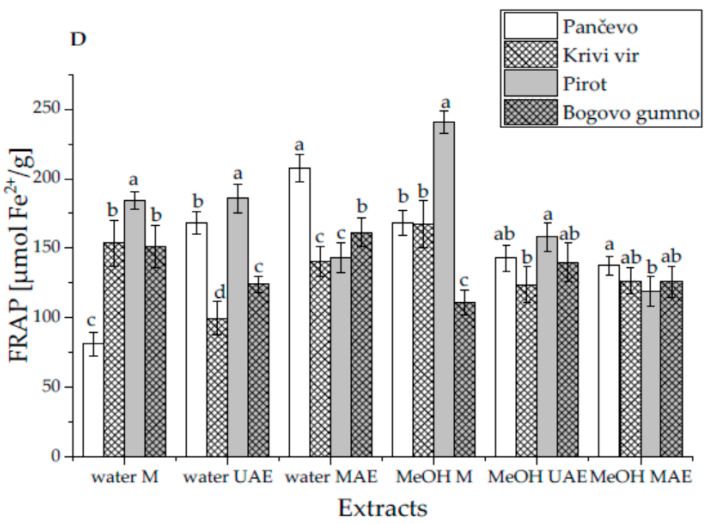
Antioxidant potential (**A**) ABTS, (**B**) DPPH, (**C**) CUPRAC, and (**D**) FRAP assays of extracts prepared using *Paeonia peregrina* Mill. petals from different localities in Serbia (Pančevo, Krivi vir, Pirot, and Bogovo gumno), different extraction mediums (water and methanol, MeOH), and different extraction techniques (maceration—M, ultrasound-assisted extraction—UAE, and microwave-assisted extraction—MAE); values with the same letter in each group (the same extraction medium and technique) showed no statistically significant difference between different regions (*p* > 0.05; n = 3, one-way ANOVA, analysis of variance, Duncan’s post hoc test).

The impact of the origin of the petals of *P. peregrina*, the extraction method, and the extraction medium on the antioxidant capacity of the extracts was estimated using ABTS, DPPH, CUPRAC, and FRAP assays, and the outcomes are presented in Figure 6. The impact of a single factor (location, technique, or medium) is presented in Supplementary Figures S4 and S5.

#### 2.4.1. ABTS-Radical-Scavenging Activity of the Extracts

According to the results presented in Figure 6A, there were no differences between the extracts obtained by maceration and UAE regarding their origin, except for the locality Krivi vir (water extract), which showed lower ABTS-radical-scavenging activity. For MAE, the water extract from Pirot and the methanol extract from Bogovo gumno showed the lowest antioxidant potential (Figure 6B). The origin of the plant material had no effect on the ABTS-radical-scavenging activity (Supplementary Figure S4A). Furthermore, neither the extraction technique nor the medium had any effect on the antioxidant capacity of the extracts (Supplementary Figure S4B,C, respectively).

The ABTS antioxidant capacity results did not follow the TPC and TFC trends, which can be explained by the fact that various plant metabolites (other than polyphenols and their subgroup, flavonoids), as well as their interactions, may significantly impact the overall antioxidant potential of the extracts. This phenomenon was also observed in a study on *T. serpyllum*, in which no differences in ABTS antioxidant activities were observed between the extracts prepared using traditional extraction technique (maceration) or novel extraction techniques (heat-assisted extraction, UAE, etc.) [54]. The degradation of sensitive antioxidant compounds in the presence of ultrasound waves (an ultrasound probe) and high temperatures (in a microwave reactor) is a possible explanation for the weak correlation between the ABTS and TFC values, as it was also observed in a study with red rose petals [36]. Additionally, free radicals can be generated by the ultrasound probe, consequently decreasing the overall antioxidant capacity of the extracts. From the standpoint of the extraction medium used, since most biologically active compounds in *P. peregrina* petals are polar molecules (flavonoid glycosides, anthocyanins, terpenes, phenolic acids, etc.), methanol and water are considered suitable extraction mediums. Similarly, the ABTS radical scavenging capabilities of the water and methanol extracts of *Melissa officinalis* did not differ from each other [37].

#### 2.4.2. DPPH-Radical-Scavenging Activity of the Extracts

The concentrations of all the studied *P. peregrina* extracts required to scavenge 50% of the free radicals (DPPH IC_50_) were in the range of 0.110 to 0.125 mg/mL (Figure 6B). When using the maceration technique, there was no difference between the extracts from different localities, except in the case of Bogovo gumno (water extract), which showed a higher IC_50_ value, implying a lower DPPH-radical-scavenging capacity. For UAE, the water extract from Krivi vir and the methanol extracts from Krivi vir and Pirot showed higher activity then those from other localities (Figure 6B); meanwhile, for MAE, both the water and methanol extracts from Pirot showed the highest DPPH antioxidant activity (Figure 6B). As was the case for the ABTS assay, neither the origin of the petals nor the extraction method affected the extracts’ ability to scavenge DPPH radicals (Supplementary Figure S4A,B). In contrast to the ABTS assay, the DPPH antioxidant activity of the extracts was significantly influenced by the extraction medium, favoring methanol (Supplementary Figure S4C). This can be explained by the reactivity of free radicals and their different mechanisms of reaction. Specifically, ABTS radicals are more reactive compared to DPPH radicals. DPPH radicals are involved in the transfer of hydrogen atoms, while ABTS radicals react via electron transfer. Therefore, the higher reactivity of the ABTS radicals resulted in the high radical-scavenging potential of the water and methanol extracts, although the water extracts possessed significantly lower TPC and TFC. On the other hand, in the DPPH assays, methanol extracts with significantly higher polyphenol and flavonoid concentrations exerted higher antioxidant potential. The higher solubility of some flavonoid derivatives (including flavanones and phenylpropanoids) in the methanol extracts, compared to their water parallels, lent them better biological efficacy [55]. In a study with extracts of red rose petals, the samples with higher flavonoid content showed stronger DPPH-radical-scavenging activity [36]. The reduction in DPPH radicals might be associated with the reducing activity of flavonoid compounds [56]. Nevertheless, some flavonoids have antioxidant potential but cannot be measured because they lack the necessary chelating functional groups for Al^3+^; thus, they are unable to react with aluminum chloride [29].

#### 2.4.3. Cupric-Ion-Reducing Antioxidant Capacity of the Extracts

When using maceration and UAE, the water extracts from Pančevo and Krivi vir and the methanol extractd from Pančevo had the highest cupric-ion-reducing potential, while, for MAE, the same was observed with both the methanol and water extracts from Pančevo and the water extract from Bogovo gumno (Figure 6C). While the extraction method and medium did not impact the cupric ion, the origin of the plant material influenced it significantly (Supplementary Figure S5). The extracts with the highest antioxidant potential were made using the petals from Pančevo, confirming that the chemical composition of plant material depends on its geographic origin. This is in agreement with a study on *Rosa damascene*, in which the origin of the petals significantly influenced the cupric-ion-reducing capacity of the extracts [57].

According to Özyürek et al. [58], cupric ions can be reduced by various types of antioxidant compounds (including polyphenols, flavonoids, tannins, thiols, D-ascorbic acid, mannitol, glucose, etc.) but some other organic components (plant pigments, free organic acids, and proteins) can intensify the aforementioned reaction.

#### 2.4.4. Ferric-Reducing Antioxidant Power of the Extracts

When using both the maceration and UAE techniques, the water and methanol extracts of the petals from Pirot demonstrated the highest ferric-reducing antioxidant power, while their counterparts obtained through MAE possessed the lowest antioxidant potential (Figure 6D). On the other hand, for MAE, the water and methanol extracts from Pančevo had the highest ferric-reducing activity. As already confirmed by the CUPRAC assay, the origin of the *P. peregrina* petals significantly affected the ferric-reducing capacity of the extracts (Supplementary Figure S5A). The other two variables (the extraction procedures and mediums) did not show the same effect (Supplementary Figure S5B,C, respectively).

Petkova et al. [57] also reported that the origin of rose petals affected the ferric-reducing antioxidant power of the extracts, and that the best results in the CUPRAC and FRAP tests differed. The extract prepared using rose petals from one region possessed the highest value for ferric-reducing potential but not the highest value for cupric ion reduction, as was the case in our study. The discrepancy observed when comparing different extraction factors is not surprising given that the oxidation probes, targeted compounds, mechanism, and kinetics of the reaction, as well as the conditions during the assays (pH, measurement wavelength, and time), differed between all of the antioxidant tests performed.

### 2.5. Antimicrobial and Antibiofilm Activities

As plant material from various origins may differ in terms of its chemical composition, specifically, in terms of the bioactive constituents responsible for antimicrobial effects, the extracts prepared from the petals of *P. peregrina* collected from four different Serbian localities were tested for their antibacterial and anticandidal activities. In Table 4, the effectiveness of the petals’ water and methanol extracts against the three bacteria *Staphylococcus lugdunensis*, *Proteus vulgaris*, and *Staphylococcus aureus* is presented.

The antibacterial activity of the petal extracts from Pančevo showed the best antibacterial activity, with the extracts being the most effective against *S. lugdunensis* (MIC 0.0625–0.125 mg/mL), followed by *P. vulgaris* and *S. aureus*, against which the extracts were equally effective. On the other hand, the extracts of the petals from Krivi vir and Bogovo gumno exhibited similar activity against all the tested bacteria, with the MIC values of both groups of extracts against *S. lugdunensis* being around 0.25 mg/mL, meaning that they are the most potent in suppressing its development. Additionally, both the methanol and water extracts of the petals from all four localities showed better activity against *S. aureus* (MIC 0.25–1 mg/mL) than the positive control, the antibiotic gentamicin (MIC 1.33 mg/mL).

The extracts were also evaluated for antifungal activity against three therapeutically important *Candida* species (Table 5). Each extract’s effectiveness was compared to that of ketoconazole, a standard antifungal drug. All the *P. peregrina* petal extracts showed antifungal activity against the tested *Candida* strains. The greatest antifungal impact, however, was observed against *C. albicans*, a significant fungal pathogen that is capable of producing invasive candidiasis in the urinary tract, skin, and/or mucus or genitals. The MIC values of the extracts against *C. kefyr* ranged from 0.25 to 4 mg/mL, whereas the MFC values ranged from 0.5–8 mg/mL. *C. krusei* had the highest resistance, with a MIC of 4 mg/mL and a MFC of 8 mg/mL.

As the extracts of petals from Pančevo obtained using methanol and UAE and maceration, those from Bogovo gumno obtained by maceration with methanol, and the petals from Pirot extracted by methanol with the use of UAE exhibited the highest inhibitory action against *S. lugdunensis*, they were chosen for further investigation of their antibiofilm activity. The findings are displayed in Table 6. The tested extracts showed a lack of potential to inhibit the formation of a bacterial biofilm, as only the methanol extract of petals from Pančevo obtained by maceration showed 14% activity at a 1/4 MIC concentration of the used extract.

Herein, water and methanol extracts of the petals of *P. peregrina* were studied as a potential source of antimicrobial agents intended for therapeutic skin treatment. As the phenolic acids represented the most abundant group of compounds in the analyzed extracts, with the dominance of gallic acid and its derivatives, which have already been confirmed to be strong antibacterial agents [32,33], such activity was expected.

Little previous work has used the microdilution method to consider the antibacterial activity of the herbaceous peony petal extracts [25]. The methanol and water extracts of the petals of *P. tenuifolia* were subjected to the antibacterial assay against the same three bacteria (*S. aureus*, *P. vulgaris*, and *S. lugdunensis*) [25]. The methanol macerate of *P. peregrina* petals from Pančevo showed significantly better antibacterial potential against *S. lugdunensis* in comparison to the results for both the methanol and water extracts of *P. tenuifolia* [25], whereas all of the extracts examined in this study are less effective in inhibiting the growth of the remaining two bacteria (*P. vulgaris* and *S. aureus*) when compared to the petal extracts from the previously mentioned work. The difference in the antibacterial activity of the petals can be attributed to the difference in the chemical composition between these two peony species.

There has been little previous research on the effects of the methanol and water extracts of petals of herbaceous peonies, with only one study conducted by our research group on the petals of *P. tenuifolia* [25]. In that study, the minimal concentration needed to inhibit the growth of *C. albicans* was 0.5 mg/mL, whereas the concentration of the extracts of *P. peregrina* petals from Krivi vir needed to inhibit the growth of the same fungi ranged from 0.125 to 0.5 mg/mL, making it four times more effective than the petals of *P. tenuifolia*. On the other hand, the concentrations needed to ensure activity against the remaining two fungi (*C. kefyr* and *C. krusei*) were in the range of 0.25 to 1 mg/mL, which is also somewhat more effective than the petals of *P. tenuifolia*.

However, compared to the results presented in the previous study [25], the extracts of *P. peregrina* demonstrated much lower antibiofilm activity than those of *P. tenuifolia*, which is associated with differences in their chemical composition.

### 2.6. Cytotoxicity

**Figure 7:**
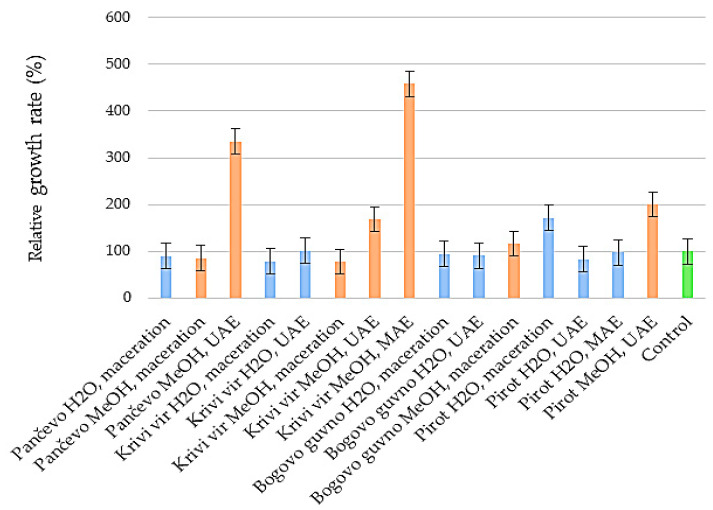
Relative growth rate of the HaCaT cell line in the presence of water and methanol extracts of the petals of *Paeonia peregrina* Mill. UAE, ultrasound-assisted extraction; MAE, microwave-assisted extraction. The relative growth rate of the HaCaT cells was determined in triplicate.

In vitro cytotoxicity and cell proliferation studies are important first steps in determining a test drug’s potential toxicity in humans, including plant extracts or biologically active compounds derived from plants [59]. All of the petal extracts were subjected to the cytotoxicity assay, and none of them had an unfavorable effect on the cell line’s growth rate (Figure 7).

After a detailed literature review, we concluded that very little work had previously been undertaken on the cytotoxicity of the petal extracts of herbaceous peonies on human keratinocytes [25], and the findings were consistent with the results presented in this study. These findings suggest that *P. peregrina* petal extracts are not toxic to skin cells and may have potential applications in the pharmaceutical, dermatological, and cosmetic industries.

### 2.7. Wound Healing

**Figure 8:**
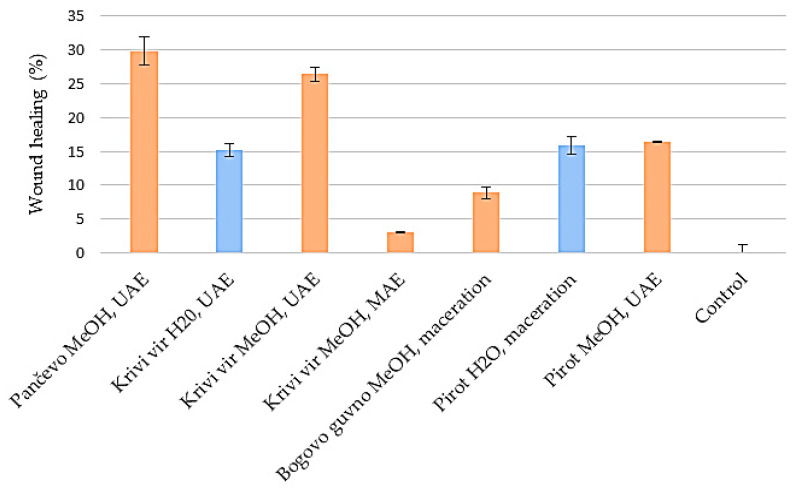
Effects of water and methanol extracts of the petals of *Paeonia peregrina* Mill. On the migration capacity of HaCaT cells (wound healing). UAE, uUltrasound-assisted extraction; MAE; microwave-assisted extraction. The effect of the petal extract on wound healing potential was assessed in triplicate.

**Figure 9:**
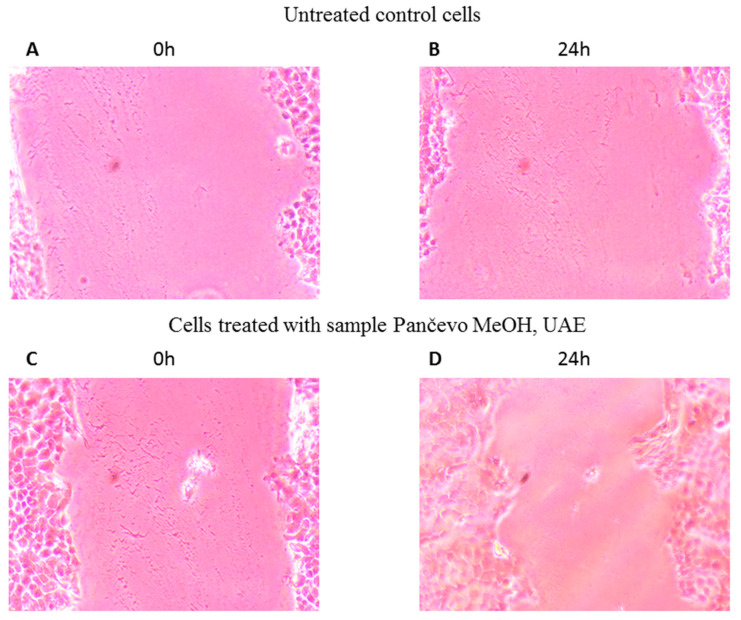
Wound healing properties of the sample MeOH, UAE; (**A**) untreated control cells at 0 h, (**B**) untreated control cells after 24 h, (**C**) cells treated with the extract at 0 h, and (**D**) cells treated with the Pančevo MeOH, UAE extract after 24 h. Ultrasound-assisted extraction (UAE); methyl alcohol (MeOH). The magnification of the microscope is 20 times.

Wound healing is an important component of the skin’s defensive and protective activities [60]. Based on the results of keratinocyte proliferation under the impact of petal extracts (Figure 7), only those with a greater rate of cell growth than the control were included in the wound healing experiment. Figure 8 and Figure 9 compare the impact of the extracts on wound closure to the control (an untreated wound).

When a methanol UAE extract of *P. peregrina* petals originating from Pančevo was applied to HaCaT cells with scratched wound gaps, the wound gap closure improved significantly compared to the control. In the case of the water extracts, both the Krivi vir and Pirot petal extracts exhibited similar effectiveness in assisting wound closure (15.19% and 15.92%, respectively). In terms of the remaining extracts’ efficacy (Figure 8), the UAE methanol extract of the petals from Krivi vir outperformed the control, exceeding the MAE methanol extracts from the same locality, as well as the methanol macerate of the petals from Bogovo gumno and the UAE methanol extract of the petals from Pirot.

To the best of our knowledge, these are the first findings providing proof that *P. peregrina* petal extracts enhance keratinocyte migration and proliferation. Because of their antioxidant and antibacterial qualities, phenolics, flavonoids, and terpenoids are known to aid wound healing. These properties appear to be responsible for wound contraction and an increased rate of epithelialization [61]. Hence, our findings indicate that *P. peregrina* petals may aid in healing skin wounds. To the best of the authors’ knowledge, extracts of the petals of herbaceous peonies have been tested for their effects on wound healing on HaCaT cells only as a part of our previous study [25]. In comparison with the results from the aforementioned study, those presented in the present study show that the UAE methanol extract of petals from Pančevo exhibited a somewhat higher efficacy in comparison with all of the tested extracts of *P. tenuifolia* petals, which could be associated with the difference in the chemical composition of the petals.

### 2.8. Anti-Inflammatory Activity

The potential of the petal extracts to suppress the denaturation of protein was explored as part of the inquiry into the mechanism of anti-inflammatory action. The inhibitory effects of various concentrations of the three *P. peregrina* petal extracts with the highest polyphenol content on protein denaturation are presented in Table 7. *P. peregrina* petal extracts (250–1000 µg/mL) achieved the significant inhibition of the denaturation of bovine serum albumin (BSA), in a dose-dependent manner. The in vitro anti-inflammatory activities of the extracts were comparable to those of ibuprofen, a reference drug (25–100 µg/mL). A significant difference in the inhibition of thermally induced protein denaturation was observed between the extracts and ibuprofen at the highest tested concentration, 100 µg/mL.

At 1000 μg/mL, the maximum inhibition was 62.22% (Table 7) for the methanol MAE extract of petals from Pančevo, which was three times more effective than ibuprofen at the highest tested concentration (100 μg/mL). The methanol macerate of the petals from Pančevo and the MAE extract of petals from Krivi vir were somewhat less effective (53.75 and 47.04%, respectively). This phenomenon may be associated with the polyphenol content of the extracts, making them more effective when they are richer in phenolic compounds.

To the best of our knowledge, this was the first study in which the anti-inflammatory activity of the petal extracts of herbaceous peonies was assessed. On the other hand, the petal extracts of *Wedelia trilobata* [62] were examined on the basis of halting heat-induced protein denaturation, and the extracts showed a 50% lower ability to slow down the degradation of protein in comparison to the petals of *P. peregrina*, which we can assume to be due to the difference in the plant species that were examined.

## 3. Materials and Methods

### 3.1. Origin of Plant Material

**Figure 10:**
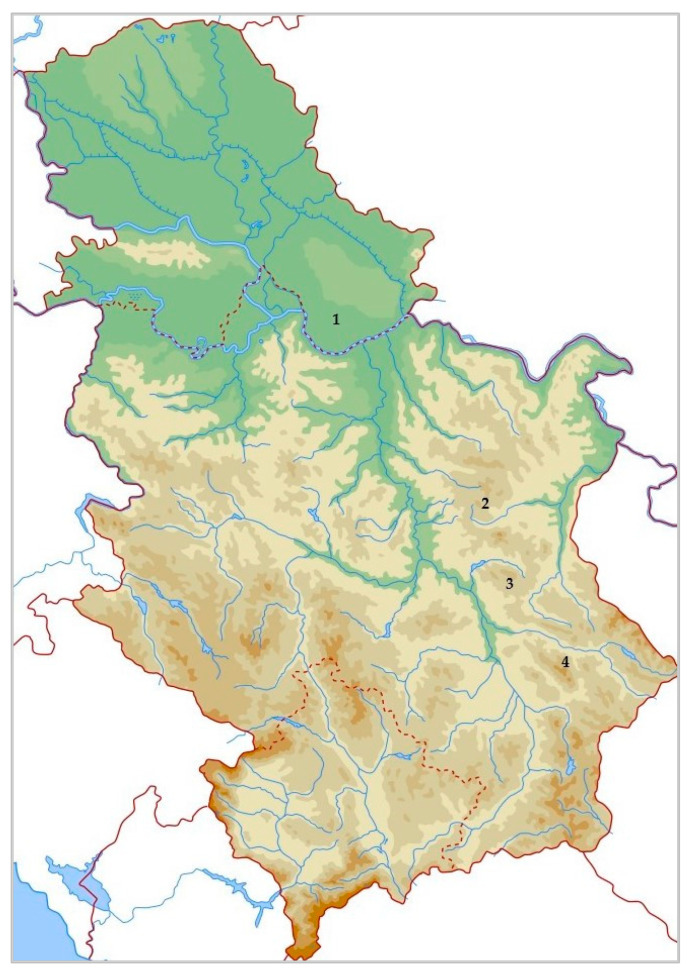
Localities in Serbia where *P. peregrina* petals were collected; (1) Pančevo; (2) Krivi vir; (3) Pirot; and (4) Bogovo gumno.

Fresh petals of *Paeonia peregrina* Mill. were collected in May 2022 from plants growing spontaneously in their natural habitats in Serbia, specifically, in Krivi vir at 467 m a.s.l. (16 May), Bogovo gumno at 952 m a.s.l. (12 May), and Pirot at 666 m a.s.l. (23 May), as well as from the previously domesticated plants in the Institute’s collection in Pančevo at 100 m a.s.l. (18 May) (Figure 10).

The collection of the petals from the wild was conducted with the permission of the Ministry of Environmental Protection of the Republic of Serbia (No. 353-01-162/2022-04, issued on 24 February 2022). The collection of petals was performed manually from randomly selected full-blooming plants. At each locality, 1/3 of the petals per flower were collected from less than 10% of the flowering plants found.

Following collection, the petals were kept in paper bags in a dark and cold place until the extractions were performed (the day after collection). Voucher specimens of this strictly protected plant species in Serbia were confirmed and deposited at the Herbarium BUNS at the Department of Biology and Ecology, Faculty of Sciences, University of Novi Sad, Serbia: (1) *Paeonia peregrina* Mill., Krivi vir, Serbia, BUNS 2-1103; (2) *P. peregrina* Mill., Bogovo gumno, Serbia, BUNS 1-1109; (3) *P. peregrina* Mill., Pirot, Serbia, BUNS 2-1108 (4) *P. peregrina* Mill, Pančevo, Serbia, BUNS 2-1104.

### 3.2. Extraction of Plant Material

With the aim of avoiding the influence of different percentages of water in fresh petals, the number of petals from each locality was measured before the extraction, resulting in the same solid-to-solvent ratio (1:20).

#### 3.2.1. Maceration

The petals were extracted by maceration using a linear mechanical homogenizer (Roller mixer SRT6, IKA, Königswinter, Germany) at 25 ± 5 °C for 24 h, using methyl alcohol (methanol) or distilled water as the extraction medium. The extracts prepared using petals (5 g) and the extraction medium (100 mL) were filtered using laboratory filter paper. The collected analyte was stored in a dark container at 4 °C until further analysis.

#### 3.2.2. Ultrasound-Assisted Extraction (UAE)

The ground petals (2.5 g) were extracted for 10 min at 60 °C using a 750 W ultrasonic processor with a 20 kHz converter and a solid titanium probe with a 13 mm diameter and a 70% amplitude (Cole-Parmer Ultrasonic Processor Stainless Steel Temperature Probe, Saint Neots, UK). The same extraction medium and solid-to-solvent ratio (1:20) were used as in the case of maceration. After filtering the mixture with laboratory filter paper, the raw extracts were collected and kept at 4 °C for future analysis.

#### 3.2.3. Microwave-Assisted Extraction (MAE)

MAE was performed in a Microwave Synthesis Reactor (Monowave 300, Anton Paar, Ostfildern, Germany) under the operational conditions presented in Supplementary Figure S1, using 0.5 g of the powdered samples and 10 mL of methyl alcohol (methanol) or water at 60 °C for 10 min. The extracted petals were filtered through qualitative laboratory filter paper and stored at 4 °C until further examination.

### 3.3. Chemical Analysis

#### 3.3.1. Chemicals

The following reagents were used in this study: Folin–Ciocalteu reagent, 2,2-diphenyl-1-picrylhydrazyl (DPPH), potassium ferricyanide, gallic acid, catechin, trolox, iron(II)sulfate, tryptic soy broth, ketoconazole, streptomycin, phosphate-buffed saline (PBS), and iron(III)chloride were purchased from Sigma Aldrich (St. Louis, MO, USA); sodium-carbonate from FHI Zdravlje AD Leskovac (Serbia); sodium nitrite from Alkaloid Skopje (Skopje, Macedonia); aluminum chloride, and tri-chloroacetic acid from Kemika (Zagreb, Croatia); sodium hydroxide from NRK Inzenjering (Belgrade, Serbia); 2,2’-azino-bis(3-ethylbenzothiazoline- 6-sulfonic acid)-ABTS from Roche Diagnostics GmbH (Penzberg, Germany); neocuproin from Acros Organics (Geel, Belgium); monosodium phosphate and disodium phosphate from Merck (Boston, MA, USA); cuprum chloride from Fluka (Buchs, Switzerland); ammonium acetate and ethanol from Zorka Pharma-Hemija (Šabac, Serbia); gentamicin from Panfarma (Belgrade, Serbia); crystal violet from Bio-Merieux (France); methanol and ferrous sulfate from Zorka Šabac, Serbia; high-glucose Dulbecco’s Modified Eagle Medium (DMEM) supplemented with 10% fetal bovine serum (FBS); 2 mM L-glutamine from Thermo Fisher Scientific (Altrincham, UK); and penicillin as bought from Panfarma, Šabac, Serbia.

#### 3.3.2. Determination of the Content of Active Constituents in the Extracts

##### Total Polyphenol Content

The total polyphenol content (TPC) of the petal extracts was determined using a modified Folin–Ciocalteu technique developed by Park [63]. The TPC was determined using the Folin–Ciocalteu reagent and spectrophotometric analysis. In summary, a 2000 µL flask was filled with 20 µL of sufficiently diluted extract, 100 µL of the Folin–Ciocalteu reagent, and 1500 µL of deionized water. After 5 min, 300 µL of sodium carbonate (20% *w*/*v*) was added, followed by the addition of 2000 µL of deionized water. The absorbance at 765 nm was measured using a 1800 UV/Vis spectrophotometer (Shimadzu, Kyoto, Japan) after 120 min of incubation in the dark at room temperature. The calibration curve was made with a gallic acid solution (100–800 mg/L). The results are presented as milligrams of gallic acid equivalent/g of fresh plant material (mg GAE/g). Each analysis was repeated three times, and the findings were statistically analyzed.

##### Total Flavonoid Content

The total flavonoid content (TFC) in the petal extracts was measured using a modified approach described by Park et al. [63]. Briefly, 1250 µL of deionized water was mixed with 250 µL of properly diluted extract and 75 µL of 5% sodium nitrite solution. After that, the mixture was kept in the dark for 5 min. The mixture was then treated with 150 µL of 10% aluminum (III) chloride solution and 500 µL of 1 M sodium hydroxide before being topped off with deionized water to a final volume of 2000 µL. The absorbance of the samples was measured at a wavelength of 425 nm. Each test was run in triplicate. Catechin monohydrate was used to create the calibration curve (37.5–300 mg/L). The results are expressed in milligrams of catechin equivalent (CE)/g of fresh plant material (mg CE/g).

#### 3.3.3. UHPLC-LTQ-Orbitrap MS

For the LC/MS analysis (Thermo Fisher Scientific, Bremen, Germany), an LTQ OrbiTrap XL mass spectrometer linked to an Accela 600 UHPLC system running in the positive and negative ionization mode (heated electrospray ionization or HESI) was used. For separation, a Syncronis C18 analytical column (50 × 2.1 mm, 1.7 μm particle size) was used with a gradient of two mobile phases (0.1% HCOOH in ultrapure water and 0.1% HCOOH in acetonitrile MS grade). Prior reports on UHPLC conditions and MS parameters are provided in Zengin et al. [64]. The molecule editor program ChemDraw (version 12.0, CambridgeSoft, Cambridge, MA, USA) was used for the structure drawings and for calculating the precise masses of the compounds of interest.

Xcalibur software (ver. 2.1, Thermo Fisher Scientific, Waltham, MA, USA) was used for the instrument control and data analysis. Some of the compounds for which no standards were available were tentatively identified using previously reported MS fragmentation data [65,66].

Chemical profiling of the methanol extracts of the petals was assessed using an advanced LC/MS method (UHPLC–LTQ–Orbitrap–MS). The deprotonated molecule mass [M−H]^−^ and MS^2^, MS^3^, and MS^4^ fragmentation behaviors were used for the identification of compounds in the extract, with the assistance of the data available in the literature.

#### 3.3.4. Antioxidant Assay

In contrast to assays that quantify antioxidant activity as a percentage decrease in absorbance, antioxidant activity in this study was expressed as the mol of trolox equivalents (TE)/gram of fresh plant material (CUPRAC assay), μmol Fe^2+^/g of fresh plant material (FRAP assay), mmol of trolox equivalent (TE) gram of fresh plant material, and as a half-maximal inhibitory concentration, i.e., IC_50_ (DPPH assay). This enables a more straightforward and direct comparison of antioxidant activity.

##### Cupric-Ion-Reducing Antioxidant Capacity Assay

The reaction mixture was made by mixing 0.8 mL of the extract with 1 mL of CuCl_2_x2H_2_O (copper(II) chloride dihydrate), 1 mL of neocuproine, and 1.2 mL of ammonium acetate buffer (pH ≈ 7). The sample was then incubated for 30 min at room temperature in complete darkness before the absorbance at 450 nm was measured on a UV Spectrophotometer UV-1800 (Shimadzu, Japan). For each extract, the assay result was verified three times. Trolox was used to obtain the calibration curve for this methodology, with the concentration range from 0.125 mg/mL to 1 mg/mL. In terms of TE, the obtained values are given as mol TE/g of fresh plant material.

##### Ferric-Reducing Antioxidant Power Assay

The protocol for the ferric-reducing antioxidant power (FRAP) assay used in this study was previously described by Prior et al. [67]. The petal extract (10 mg) was mixed with 1 mL of the K_3_Fe(CN)_6_ solution and 1 mL of phosphate buffer (pH ≈ 6.6), and the mixture was incubated for 4 h at 50 °C. Following the incubation period, 0.25 mL of a 10% trichloroacetic acid solution was mixed with 0.5 mL of the prepared sample. Next, 0.75 mL of distilled water and 0.17 mL of FeCl_3_ (0.1% *m*/*v*) were added. All of the reagents (without the extract) were present in the negative control. Three parallel runs of the experiment were conducted, and the absorbance was measured at 750 nm. The results are given as μmol Fe^2+^/g of fresh plant material and were calculated using ferrous sulfate to create the calibration curve (10–50 mg/L).

##### ABTS Assay

The analytical protocol described by Prior et al. [67], with modifications [68], served as the foundation for the ABTS^●+^ scavenging assay. Specifically, 200 μL of the petal extract and 2800 μL of the ABTS^•+^ radical cation solution were combined and incubated at 25 ± 5 °C in the dark for 30 min. The ABTS^•+^ radical solution (7.8 mmol/L) was made by dissolving 20 mg of ABTS_(s)_ in 5 mL of deionized water and then adding 88 μL of potassium-persulfate_(aq)_ solution with a concentration of 2.45 mmol/L (380 mg of potassium-persulfate_(s)_ was dissolved in 10 mL of deionized water). Prior to use, the ABTS stock solution mixture was incubated for 16–20 h at 4 °C in the dark to create (activate) the radical cation solution (ABTS^•+^). Following activation, the ABTS^•+^ radical cation solution was diluted by methanol or distilled water, yielding an initial absorbance of 0.70 ± 0.02, at a wavelength of 734 nm. The control solution (blank) was made by mixing 2800 µL of the ABTS^•+^ radical cation solution with 200 µL of the extraction medium in place of the extract. A triple of each measurement was made. The absorbance of each sample was measured at 734 nm. The radical scavenging activity was calculated according to following equation (Equation (1)):

(1) $$\Delta A=A_{cont}-A_{sample}\;,$$

where A_cont_ is the absorbance of the ABTS^•+^ solution and the extraction medium, while A_sample_ is the absorbance of the ABTS^•+^ solution and the extract. Trolox was used as a standard for the calibration curve. The scavenging capacity is expressed as mmol TE/g of fresh plant material (mmol TE/g).

##### DPPH Assay

The DPPH solution was created by combining 9 mL of ethyl alcohol with 0.252 mg of DPPH. The petal extract (200 μL) was combined with 2.8 mL of this solution, which was then left at room temperature for 30 min, without any illumination. After the absorbances were measured at 517 nm, the scavenging activity (SC_DPPH_) was calculated using the following equation (Equation (2)):

(2) $$SC_{DPPH}=\frac{A_{cont}-A_{sample}}{A_{cont}}\times 100\%\;,$$

where A_cont_ represents the absorbance value of the DPPH solution and the extraction medium, while A_sample_ is the absorbance of the extract sample treated with the DPPH^●^ radical. The results are presented as IC_50_ (mg/mL), the concentration of the extract required to neutralize 50% of DPPH^●^ radicals, which were calculated from the calibration curve prepared from five different concentrations of the extract and the percentage inhibition of the DPPH radicals.

#### 3.3.5. Determination of the Antimicrobial and Antibiofilm Activities of the Extracts

##### Antibacterial Activity

The antibacterial efficacy of the extracts was evaluated against Gram-positive bacteria (*Staphylococcus aureus* ATCC 11632 and *Staphylococcus lugdunensis* Ibis 2996), and Gram-negative bacteria (*Proteus vulgaris* IBR P004). The microdilution technique (96-well microtiter plates) was used to estimate the minimum inhibitory concentration (MIC) and minimum bactericidal concentration (MBC), as reported before [23]. The extracts were diluted in 30% ethanol before being added to a tryptic soy broth (TSB) medium and infected with bacteria at a final concentration of 1 × 10^6^ colony-forming units (CFU) per well. Gentamicin was added as a positive control. The MIC and MBC values are provided in milligrams per milliliter (mg/mL).

##### Antifungal Activity

The strains used for testing the anticandidal activity of the extracts were *Candida albicans* ATCC 10231, *Candida kefyr* (Y289), and *Candida krusei* (Y454). The anticandidal assay was conducted using the modified EUCAST procedure (EUCAST, 2002), as previously described [69]. The lowest concentrations of the extracts that did not induce the microscopically visible growth of tested strains were considered to be MIC. Minimum fungicidal concentrations (MFC) were determined as the lowest concentrations of the extracts, after serial sub-cultivation of 10 μL of the samples at 37 °C for 24 h, at which there was no visible growth of the tested strains, indicating the 99.5% killing of the original inoculum. Untreated yeast cells were used as controls, while ketoconazole was used as a positive control.

##### Bacterial Biofilm Inhibitory Activity

The effects of the four different extracts that showed the highest antibacterial activity against the growth of *S. lugdunensis* were subjected to a biofilm assay with minor alterations, as previously described in Smiljkovic et al. [70]. In order to create a biofilm, *S. lugdunensis* was cultured in Triptic soy broth with 2% glucose in 96-well microtiter plates with adhesive bottoms (Sarstedt, Germany) with the MIC, 1/2 MIC, and 1/4 MIC of the extracts at 37 °C for 24 h. Following the incubation, the wells were twice rinsed with sterile phosphate-buffered saline (PBS). After that, the biofilm was fixed with methyl alcohol (methanol) and air-dried, and each well was rinsed twice with PBS. Crystal violet was used to stain the biofilm for 30 min. After incubation, the crystal violet had been eliminated, the wells were cleaned with water and dried by air, and then 96% ethanol was used. Thermo Scientific’s Multiskan FC Microplate Photometer was used to measure absorbance at 620 nm. The following equation (Equation (3)) was used to determine the percentage of biofilm destruction:

(3) $$Biofilm\;destruction\left(\%\right)=\frac{A_{\left(control\right)\;}-A_{\;sample}}{A_{\;control}}\times 100\;\%$$

where A_cont_ represents the absorbance value of the blank untreated biofilm at the wavelength of 620 nm, while A_sample_ is the absorbance of the biofilm treated with the extract at the same wavelength.

#### 3.3.6. Determination of the Cytotoxicity of the Extracts

The extract’s activity was evaluated using the HaCaT cell line acquired from AddexBio (No. T0020001). The cytotoxic activity of petal extracts on a spontaneously immortalized keratinocyte (HaCaT) cell line was evaluated using the crystal violet method, as previously described by Stojković et al. [71]. PBS was used to dilute the extracts to a final concentration of 8 mg/mL. The HaCaT cells were cultured at 37 °C in a 5% CO_2_ incubator in high-glucose Dulbecco’s Modified Eagle Medium (DMEM) supplemented with 10% fetal bovine serum (FBS), 2 mM L-glutamine, 1% penicillin, and streptomycin. For 48 h, cells were planted in a 96-well microtitre adhesive plate. After the medium was removed, a range of extract dilutions was applied to the cell lines for 24 h in triplicate wells (two times), as follows: 400 > 200 > 100 > 50 > 25 > 12.5 μg/mL. The cytotoxic activity of the extracts on the cell line was categorized using the criteria listed below, which were laid out by Stojković et al. [72] IC_50_ ≤ 20 g/mL = highly cytotoxic; IC_50_ from 31 to 200 g/mL = moderately cytotoxic; IC_50_ from 201 to 400 g/mL = weakly cytotoxic; and IC_50_ > 401 g/mL = non-cytotoxic.

After removing the medium, the cells were washed twice with PBS before being stained with a 0.4% crystal violet staining solution for 20 min at room temperature. The cells were then rinsed in a stream of tap water to remove the crystal violet staining solution and air dried at room temperature. In a plate reader, the absorbance was measured at 570 nm. The results are presented as the relative growth rate (%) and compared to an untreated control (100% growth).

#### 3.3.7. Scratch Wound Healing Assay

As per Stojkovic et al. [72], the test was carried out with a few modifications. HaCaT cells were grown to confluency. The cell monolayer was scraped using a 200 µL tip. The floating cells were washed and grown in DMEM with 400 µg/mL of the extracts, since the chosen extracts did not express any cytotoxicity but exhibited a higher proliferation rate when compared to the untreated control. This DMEM was supplemented with 1% FBS, 2 mM L-glutamine, and 1% antibiotic–antimycotic, and cell migration was studied using a Nikon Eclipse TS2 microscope (The Netherlands) 24 h after the wound was established. As a control, untreated cells were used. The percentages of wound closure during exposure to the extract were utilized to present the results. Three independent tests were carried out.

#### 3.3.8. Anti-Inflammatory Activity of the Extracts According to the Inhibition of BSA Denaturation

The anti-inflammatory activity of the obtained extracts was studied by using the inhibition of albumin denaturation technique, according to Mizushima et al. [73] and Sakat et al. [74], with minor modifications. The reaction mixture consists of test compounds and a 1% water solution of a bovine serum albumin (BSA) fraction, at pH 6.4. The reaction mixture’s pH was adjusted using small amounts of 1 N HCl. The sample extracts were incubated at 37 °C for 20 min and then heated to 70 °C for 20 min; after cooling the samples, the turbidity was measured at 660 nm. The percentage inhibition of protein denaturation was calculated as follows:

$$Percentage\;of\;inhibition=\frac{\left(A_{cont}-A_{sample}\right)\;}{A_{cont}}\times 100\%$$

with reference to the correlation coefficient value (r) of 0.946, where A_cont_ stands for the absorbance value of the BSA treated with the ibuprofen sample at 660 nm, while A_sample_ is the absorbance of the BSA treated with the extract at the same wavelength. Each experiment was run in triplicate, and the average was calculated.

### 3.4. Statistical Analysis

The statistical analysis was performed using one-way analysis of variance (one-way ANOVA) and Duncan’s post hoc test in the software STATISTICA 7.0. The differences were deemed statistically significant at *p* < 0.05, n = 3. The experimental design (full factorial 23 design) was used for the determination of the level of all factors employed to obtain the extracts with the highest TPC. The experimental design method was used for the screening and optimization of process factors. Based on the results obtained in the one-way ANOVA, a 2^3^ full factorial design (two levels of three factors) was employed to investigate the effect on the TPC (dependent variable) and choose the locality (1), extraction technique (2), and extraction medium (3) for achieving the highest polyphenol yield in petal extracts of *P. peregrina*. Each factor was tested at the two most promising levels chosen based on preliminary screening.

## 4. Conclusions

This study determined the chemical makeup of *P. peregrina* petal extracts and their potential biological benefits for human skin. The findings revealed that the petals originating from Pančevo (South Banat district, Serbia) exhibit the highest total phenolic content, making them the most biologically active in multiple assays, including wound healing, antimicrobial, and antibiofilm activities, as well as antioxidant activity, as determined by the CUPRAC and ABTS tests. While the most effective extraction technique and extraction medium varied across the tests, the methanol macerate of the Pančevo petals consistently yielded favorable results.

The study sheds light on the chemical composition and biological benefits of *P. peregrina* extracts, particularly the petals from Pančevo, Serbia. The potential of *P. peregrina* to be applied in the cosmetic and pharmaceutical industries can be fully realized through deeper research into individual bioactive compounds and clinical studies using the petals of cultivated *P. peregrina* plants from South Banat, thus supporting the conservation of this critically endangered and valuable plant species.

## Figures and Tables

**Table 1:** Statistical analysis of the optimization of the extraction of polyphenols (TPC) from *Paeonia peregrina* Mill. petals using a 2^3^ factorial design.

	Effect	Std. Err.	Effect Estimates	Coeff.	Std. Err. Coeff.	*p*
TPC * (mg GAE/g)						
Constant				25.732	0.123	0.000
Main factors						
Locality (1)	−2.158	0.246	16.555	−1.079	0.123	0.000
Extraction technique (2)	4.830	0.246	−17.794	2.415	0.123	0.000
Extraction mediums (3)	8.718	0.246	13.601	4.359	0.123	0.000
Interaction of two factors						
1 by 2	2.967	0.246	−6.221	1.483	0.123	0.000
1 by 3	−2.002	0.246	0.484	−1.000	0.123	0.000
2 by 3	−0.833	0.246	−0.619	−0.417	0.123	0.003

* TPC, total polyphenol content; GAE, gallic acid equivalent.

**Table 2:** Experimental design (2^3^ factorial design) for the screening of factors’ influence on the total polyphenol content (TPC) in *Paeonia peregrina* Mill. petal extracts, with the observed and predicted values.

Locality	Extraction Technique	Extraction Medium	Locality	Extraction Technique	Extraction Medium	TPC (mg GAE */g)	
Design			Factor levels			Observed	Predicted
−1	−1	−1	Pančevo	M	Water	19.77 ± 0.39	20.10
−1	1	−1	Pančevo	M	Me-OH	31.99 ± 0.28	31.66
1	−1	1	Pančevo	MAE	Water	23.14 ± 0.49	22.80
1	1	1	Pančevo	MAE	Me-OH	32.35 ± 0.48	32.69
−1	−1	1	Pirot	M	Water	17.32 ± 0.33	16.98
−1	1	1	Pirot	M	Me-OH	24.19 ± 0.54	24.53
1	−1	−1	Pirot	MAE	Water	25.27 ± 0.52	25.61
1	1	−1	Pirot	MAE	Me-OH	31.83 ± 0.60	31.49

* GAE, gallic acid equivalent; Me-OH, methanol.

**Table 3:** HRMS and MS^4^ data for metabolites identified in *P. peregrina* methanolic extracts.

No.	Compound Names	*t*_R_, min	Molecular Formula, [M–H]^−^/[M+H]^+^	Calculated Mass, [M–H]^−^/[M+H]^+^	Exact Mass, [M–H]^–^/[M+H]^+^	ΔmDa	MS^2^ Fragments, (% Base Peak)	MS^3^ Fragments, (% Base Peak)	MS^4^ Fragments, (% Base Peak)
***Phenolic acids and derivatives***									
**1**	Galloyl- hexoside 1	0.57	C_13_H_15_O_10_^−^	331.06707	331.06438	2.69	125(8), 151(4), **169**(100), 170(3), 193(9), 211(20), 271(38)	**125**(100)	69(55), 76(8), 79(17), **81**(100), 97(56), 107(49)
**2**	Galloyl- hexoside 2	0.87	C_13_H_15_O_10_^−^	331.06707	331.06398	3.09	125(14), **169**(100), 170(7), 193(12), 211(28), 271(59), 272(7)	**125**(100)	79(19), **81**(100), 97(66), 107(26)
**3**	Gallic acid	1.00	C_7_H_5_O_5_^−^	169.01425	169.00924	5.00	124(3), **125**(100)	51(5), 53(5), 69(17), 79(10), **81**(100), 97(91), 107(14)	NA
**4**	Digalloyl- hexoside 1	2.19	C_20_H_19_O_14_^−^	483.07803	483.07383	4.20	168(9), **169**(100), 170(6), 193(4), 271(7), 313(14), 331(25)	**125**(100)	53(30), **81**(100), 97(59)
**5**	Digallic acid 1	3.00	C_14_H_9_O_9_^−^	321.02521	321.02246	2.75	125(4), **169**(100), 170(7)	**125**(100)	67(13), 69(16), **81**(100), 97(72), 107(24)
**6**	Coumaroyl Hexaric acid	3.11	C_15_H_15_O_10_^−^	355.06707	355.06402	3.05	129(3), 147(4), 173(3), **191**(100), 192(5), 209(39), 337(7)	**85**(100), 129(5), 147(9), 173(4)	**57**(100), 61(30)
**7**	Benzoyl hexaric acid	3.11	C_13_H_13_O_9_^−^	313.05651	313.05388	2.63	129(9), 147(9), 173(7), **191**(100), 192(6), 295(13)	**85**(100), 129(8), 147(9)	**57**(100)
**8**	Galloyl- norbergenin 1	3.28	C_20_H_19_O_13_^+^	467.08202	467.08282	−0.80	**153**(100), 237(10), 279(9), 297(18), 305(27), 448(8), 449(20)	79(4), **125**(100), 143(29)	79(41), **97**(100), 107(8), 143(6), 175(3), 248(3)
**9**	Methyl gallate 1	3.34	C_8_H_7_O_5_^−^	183.02990	183.03519	−5.29	124(75), 139(4), 140(8), 153(8), **168**(100), 169(5), 183(6)	111(6), **124**(100), 137(4), 139(3), 140(11)	NA
**10**	Digalloyl- hexoside 2	3.49	C_20_H_19_O_14_^−^	483.07803	483.07378	4.25	169(11), 193(16), 211(16), **271**(100), 272(12), 313(22), 331(26)	169(12), **211**(100)	124(26), 125(8), 139(5), 165(11), 167(46), **168**(100), 183(9)
**11**	Digallic acid 2	3.49	C_14_H_9_O_9_^−^	321.02521	321.02212	3.08	125(3), **169**(100), 170(4)	**125**(100)	69(28), 79(16), **81**(100), 95(10), 96(9), 97(57), 107(15)
**12**	Benzoyl- dihexoside	3.61	C_19_H_25_O_12_^−^	445.13515	445.13137	3.78	161(59), 162(6), 179(24), 221(7), 321(10), **323**(100), 324(26)	113(73), 125(78), 143(57), 179(45), **221**(100), 245(41), 263(90)	NA
**13**	Trigalloyl- hexoside 1	3.62	C_34_H_19_O_13_^−^	635.08311	635.08409	−0.98	313(6), 421(6), **465**(100), 466(18), 483(9), 483(52), 484(9)	161(7), 169(62), 193(7), 235(10), 295(32), **313**(100), 421(50)	125(13), 137(4), 151(9), **169**(100), 179(5), 193(4), 295(13)
**14**	*p*-Coumaroyl hexoside	3.68	C_15_H_17_O_8_^−^	325.09289	325.09011	2.78	119(9), 145(5), **163**(100), 164(7)	**119**(100)	**101**(100)
**15**	HHDP-hexoside	3.90	C_21_H_21_O_13_^−^	481.09877	481.09555	3.22	315(58), 316(22), 319(37), **345**(100), 346(26), 463(25), 464(20)	137(8), **139**(100), 164(9), 165(85), 183(23), 207(9), 327(9)	70(38), 71(69), 76(23), 95(24), **97**(100)
**16**	Dihydroxybenzoic acid	3.92	C_7_H_5_O_4_^−^	153.01880	153.02423	−5.43	97(21), 108(4), **109**(100), 110(9), 111(4), 125(3), 138(4)	65(97), **66**(100), 72(93)	NA
**17**	Digalloyl- pentoside	3.99	C_20_H_19_O_13_^−^	467.08311	467.07949	3.63	313(9), 315(22), 421(8), **423**(100), 424(18), 425(3), 449(4)	125(6), 151(4), 168(5), 169(49), 211(4), **313**(100), 314(11)	125(17), 151(5), **169**(100), 211(5), 223(8), 241(4), 253(5)
**18**	Trigalloyl- hexoside 2	4.07	C_34_H_19_O_13_^−^	635.08311	635.08481	−1.69	**465**(100), 466(18), 467(4)	169(36), 193(4), 211(6), 235(8), 295(10), **313**(100), 447(4)	125(16), 151(7), **169**(100), 193(29), 241(17), 253(16), 295(15)
**19**	Galloyl- norbergenin 2	4.10	C_20_H_19_O_13_^+^	467.08202	467.08291	−0.89	**153**(100), 233(8), 261(21), 279(8), 297(20), 449(8), 450(10)	79(4), **125**(100), 143(27)	69(3), 79(70), **97**(100), 107(22)
**20**	Galloyl-HHDP- hexose 1	4.26	C_27_H_23_O_17_^+^	619.09298	619.09454	−1.57	237(9), 297(15), 304(14), **305**(100), 449(30), 600(11), 601(59)	**153**(100)	79(5), **125**(100)
**21**	Tetragalloyl- hexoside 1	4.32	C_41_H_23_O_17_^−^	787.09407	787.09506	−0.99	465(5), 617(15), **617**(100), 618(25), 635(8)	277(10), 295(23), 313(8), 447(25), 449(7), **465**(100), 573(6)	169(21), 193(4), 271(6), 295(14), **313**(100)
**22**	Trigalloyl- hexoside 3	4.34	C_34_H_19_O_13_^−^	635.08311	635.08459	−1.48	295(5), 313(19), 423(7), **465**(100), 466(19), 483(94), 484(15)	169(29), 295(14), **313**(100), 314(9)	125(18), 151(4), **169**(100), 193(4), 241(11), 253(9), 295(3)
**23**	Galloyl-methylhydroxy benzoyl-hexoside	4.37	C_21_H_21_O_12_^−^	465.10385	465.10090	2.95	169(16), 193(7), 205(6), 271(5), 295(12), **313**(100), 447(4)	NA	NA
**24**	Galloyl-HHDP- hexose 2	4.40	C_27_H_23_O_17_^+^	619.09298	619.09470	−1.72	233(7), 237(3), 243(3), 261(18), 279(8), **449**(100), 467(3)	153(100), 297(16)	79(4), **125**(100)
**25**	Digallic acid methyl ester 1	4.64	C_15_H_11_O_9_^−^	335.04086	335.03802	2.84	182(4), **183**(100), 184(5)	111(3), 124(77), 137(3), 139(4), 140(7), **168**(100)	111(4), **124**(100), 137(5), 139(4), 140(10)
**26**	Ellagic acid	4.69	C_14_H_5_O_8_^−^	300.99899	300.99682	2.17	185(53), 229(87), **257**(100), 271(66), 272(24), 284(51), 301(51)	157(4), 185(82), 201(13), 213(22), **229**(100), 230(4), 240(9)	145(11), 147(12), 157(46), 173(35), **185**(100), 201(92)
**27**	Tetragalloyl- hexoside 2	4.73	C_41_H_23_O_17_^−^	787.09407	787.09491	−0.84	465(17), 617(98), 618(22), 619(6), **635**(100), 636(27), 637(7)	**465**(100), 483(8)	169(31), 193(3), 211(4), 235(6), 295(8), **313**(100), 447(4)
**28**	Galloyl-HHDP- hexose 3	4.76	C_27_H_23_O_17_^+^	619.09298	619.09467	−1.70	237(68), 261(15), 305(34), 449(36), 600(16), **601**(100), 602(17)	237(26), 261(9), 279(8), 305(20), 448(14), **449**(100), 583(4)	**153**(100), 237(3), 261(6), 279(3)
**29**	Pentagalloyl- hexoside 1	4.78	C_41_H_31_O_26_^−^	939.11091	939.10623	4.67	617(8), 787(25), **787**(100), 788(29)	465(6), 617(13), **617**(100), 635(16)	277(11), 295(22), 313(8), 447(24), 449(4), **465**(100), 573(6)
**30**	Ethyl gallate	4.86	C_9_H_9_O_5_^−^	197.04555	197.04403	1.51	124(5), 125(8), 167(3), 168(7), **169**(100), 170(4)	**125**(100)	69(19), 79(13), **81**(100), 96(5), 97(52), 107(17)
**31**	Methyl gallate 2	4.89	C_8_H_7_O_5_^−^	183.02990	183.02216	7.74	111(4), 124(72), 137(3), 139(4), 140(7), **168**(100)	111(6), **124**(100), 127(4), 137(7), 139(6), 140(14)	NA
**32**	Pentagalloyl- hexoside 2	5.02	C_41_H_31_O_26_^−^	939.11091	939.10421	6.69	617(7), **769**(100), 770(24), 771(7), 787(7), 788(3)	429(13), 431(13), 447(25), 599(24), 601(30), **617**(100), 725(9)	271(7), 277(6), 295(5), 313(8), 423(12), 447(22), **465**(100)
**33**	Digalloyl-HHDP- protoquercitol	5.06	C_34_H_27_O_21_^+^	771.10394	771.10670	−2.77	233(34), 261(96), 279(58), 304(31), **305**(100), 413(14), 431(70)	**153**(100)	79(6), **125**(100)
**34**	Pentagalloyl- hexoside 3	5.19	C_41_H_31_O_26_^−^	939.11091	939.10522	5.69	769(4), **787**(100), 788(22)	403(4), 447(6), 465(11), 573(7), **617**(10), 617(100), 635(20)	295(15), 403(33), 421(14), 447(41), 449(11), **465**(100), 573(55)
**35**	Digallic acid methyl ester 2	5.25	C_15_H_11_O_9_^−^	335.04086	335.03822	2.63	**183**(100), 184(4)	111(4), 124(72), 137(3), 139(4), 140(7), **168**(100)	111(6), **124**(100), 127(4), 137(7), 139(6), 140(14)
**36**	Methyl gallate 3	5.25	C_8_H_7_O_5_^−^	183.02990	183.02879	1.10	111(3), 124(74), 137(4), 139(4), 140(6), **168**(100)	111(3), **124**(100), 137(7), 139(4), 140(11)	NA
**37**	Trigalloyl-HHDP- protoquercitol	5.27	C_41_H_31_O_25_^+^	923.11489	923.11888	−3.99	**305**(100), 413(19), 431(59), 457(26), 583(18), 601(28), 771(37)	**153**(100)	79(4), **125**(100)
**38**	Ethyl-digallate	5.56	C_16_H_13_O_9_^−^	349.05651	349.05390	2.60	**197**(100), 198(6)	124(4), 125(7), 168(9), **169**(100)	**125**(100)
**39**	Trihydroxybenzoyl- benzoyl-hexoside	5.75	C_20_H_19_O_11_^−^	435.09329	435.09060	2.69	150(13), 168(80), 169(74), **313**(100), 314(12), 417(90), 418(20)	125(32), 137(87), 151(21), 161(15), 168(75), **169**(100), 269(47)	108(4), 123(4), 125(62), **151**(100)
**40**	*p*-Coumaric acid	5.82	C_9_H_7_O_3_^−^	163.04007	163.03925	0.81	91(3), **119**(100), 120(10)	**91**(100), 101(74), 105(54), 161(53), 168(54), 192(49), 232(55)	NA
**41**	Trigallic acid Methyl ester	5.94	C_22_H_15_O_13_^−^	487.05181	487.04859	3.22	183(9), 334(16), **335**(100), 336(8)	**183**(100)	111(5), 124(74), 137(3), 139(3), 140(7), **168**(100)
**42**	Hydroxybenzoyl- galloyl-hexoside	5.97	C_20_H_19_O_12_^−^	451.08820	451.08493	3.27	137(8), 169(7), 313(92), 314(13), **331**(100), 332(12), 349(7)	125(34), 150(12), 167(19), 168(95), 169(29), **313**(100), 314(13)	108(48), 117(42), 125(90), 135(48), 137(31), **150**(100), 151(44)
**43**	Dihydroxybenzoyl- methylgallate	6.07	C_15_H_11_O_8_^−^	319.04594	319.04355	2.39	**183**(100), 184(7)	111(3), 124(72), 137(5), 139(4), 140(8), **168**(100)	82(3), 111(8), **124**(100), 137(6), 139(7), 140(6)
**44**	Trigalloyl- pentoside	6.16	C_27_H_23_O_15_^−^	587.10370	587.10041	3.29	**169**(100), 170(5), 417(38), 418(8), 435(41), 436(6), 465(4)	**125**(100)	51(48), 55(50), 81(63), 97(47), **107**(100)
**45**	Digallic acid methyl ester 3	6.49	C_15_H_11_O_9_^−^	335.04086	335.03801	2.84	183(20), 244(4), 261(7), 276(3), 303(7), **307**(100), 308(14)	247(73), 251(17), 260(18), 261(28), 279(33), **289**(100), 290(11)	201(7), 229(3), **261**(100), 262(9)
**46**	Phenylethanol gallate	7.54	C_15_H_13_O_5_^−^	273.07685	273.07478	2.07	125(19), **169**(100), 170(6)	**125**(100)	81(70), 83(18), **97**(100)
***Flavonoid glycosides and aglycones***									
**47**	Taxifolin 3,7-di-*O*-hexoside	3.16	C_34_H_27_O_12_^−^	627.15080	627.15153	−0.73	267(25), 285(18), 355(19), 447(49), 463(9), **465**(100), 466(18)	241(14), **285**(100), 303(29), 329(4), 339(3), 339(6)	149(16), 199(23), 217(39), **241**(100), 242(22), 243(31), 257(16)
**48**	Quercetin 3-*O*-(2″-rhamnoside)-hexoside-7-*O*-hexoside	3.52	C_33_H_39_O_21_^−^	771.19840	771.19315	5.25	299(10), 301(8), 462(28), 463(18), **609**(100), 610(57), 611(9)	255(17), 271(32), **300**(100), 301(41), 445(18), 463(11), 489(11)	151(5), 227(4), 254(11), 255(33), 256(11), **271**(100), 272(31)
**49**	Methyl (epi)catechin hexuronide	3.54	C_22_H_23_O_12_^−^	479.11950	479.11546	4.04	231(7), 295(13), 299(36), 315(7), **317**(100), 318(14), 341(47)	165(65), 193(15), 229(10), **231**(100), 273(32), 289(7), 299(6)	123(6), 174(5), 187(5), 188(5), 203(5), 215(13), **216**(100)
**50**	Quercetin 3,7-di-*O*-hexoside	3.74	C_34_H_25_O_12_^−^	625.13515	625.13602	−0.87	301(39), 302(7), 462(3), 462(24), **463**(100), 464(19), 505(4)	271(5), 299(3), 300(47), **301**(100), 343(8)	107(9), **151**(100), 179(52), 229(13), 255(16), 272(14), 273(10)
**51**	Quercetin 3-*O*-hexoside-7-*O*- pentoside	3.80	C_26_H_27_O_16_^−^	595.13046	595.12574	4.72	301(36), 302(5), **433**(100), 434(21), 462(71), 463(61), 464(11)	179(3), 271(6), **300**(100), 301(25), 343(4)	151(5), 179(3), 254(11), 255(25), **271**(100), 272(14)
**52**	Kaempferol 3-*O*-hexoside-7-*O*- pentoside	3.99	C_26_H_27_O_15_^−^	579.13554	579.13192	3.62	285(7), **417**(100), 418(20), 446(12), 447(5), 459(13)	255(9), **284**(100), 285(22), 327(11)	227(14), **255**(100), 256(21)
**53**	Kaempferol 3-*O*-(2″-hexosyl)- hexoside	4.02	C_27_H_29_O_16_^−^	609.14611	609.14187	4.24	285(24), 286(4), 327(4), **447**(100), 448(20), 489(12), 490(3)	151(4), 227(4), 255(18), 256(4), **284**(100), 285(39), 327(16)	227(16), **255**(100), 256(20)
**54**	Isorhamnetin 3-*O*-(2″-hexosyl)- hexoside	4.13	C_28_H_31_O_17_^−^	639.15610	639.15208	4.02	315(16), 316(3), 357(3), **477**(100), 478(18), 519(10)	271(10), 285(8), 286(4), 299(5), **314**(100), 315(45), 357(18)	243(33), 257(10), 271(84), **285**(100), 286(48), 299(12), 300(79)
**55**	Quercetin 3-*O*-hexoside-7-*O*- rhamnoside	4.51	C_27_H_29_O_16_^−^	609.14611	609.14187	4.24	301(51), 302(8), 446(50), **447**(100), 448(17), 463(72), 464(12)	300(6), **301**(100)	107(14), **151**(100), 179(58), 211(9), 229(13), 255(22), 273(12)
**56**	Kaempferol 3-*O*-pentoside-7-*O*- hexoside	4.60	C_26_H_27_O_15_^−^	579.13554	579.13199	3.55	301(30), 302(5), 433(79), 434(14), **446**(100), 447(85), 448(12)	271(3), **299**(100), 300(3)	227(3), 243(6), 255(6), **271**(100)
**57**	Quercetin 3-*O*-(6″-galloyl)- hexoside	4.71	C_28_H_23_O_16_^−^	615.09860	615.09548	3.12	300(5), 301(16), 302(3), **463**(100), 464(17)	300(31), **301**(100)	151(80), **179**(100), 193(6), 229(7), 257(12), 273(18), 283(7)
**58**	Quercetin 3-*O*-(2″-rhamnoside)-hexoside	4.73	C_27_H_29_O_16_^−^	609.14611	609.14246	3.65	255(10), 271(23), 299(14), **300**(100), 301(30), 445(11), 489(8)	243(4), 254(8), 255(47), 256(3), **271**(100), 272(10)	199(23), 203(9), 215(34), 227(76), 229(10), **243**(100)
**59**	Kaempferol 3-*O*-hexoside-7-*O*- rhamnoside	4.76	C_27_H_29_O_15_^−^	593.15119	593.14688	4.31	285(28), 286(4), 431(50), 432(9), **447**(100), 448(17)	151(3), 227(5), 255(17), 256(4), **284**(100), 285(28), 327(16)	227(15), **255**(100), 256(18)
**60**	Isorhamnetin 3-*O*-hexoside-7-*O*- rhamnoside	4.84	C_28_H_31_O_16_^−^	623.16176	623.15762	4.14	315(15), 316(3), 461(44), 462(7), **477**(100), 478(17)	271(7), 285(10), 286(4), 299(5), **314**(100), 315(23), 357(16)	243(31), 257(13), 271(81), **285**(100), 286(44), 299(13), 300(11)
**61**	Kaempferol 3-*O*-rhamnoside-7-*O*- pentoside	4.85	C_26_H_27_O_14_^−^	563.14010	563.13695	3.15	285(55), 286(9), 417(57), 418(8), 430(41), **431**(100), 432(16)	284(6), **285**(100)	169(62), 185(52), **213**(100), 229(68), 239(51), 243(91), 257(65)
**62**	Quercetin 3-*O*-hexoside	4.95	C_21_H_19_O_12_^−^	463.08820	463.08534	2.86	300(31), **301**(100), 302(9)	107(7), 151(81), **179**(100), 256(10), 257(11), 272(14), 273(19)	**151**(100)
**63**	Quercetin 3-*O*-pentoside	5.16	C_20_H_17_O_11_^−^	433.07764	433.07458	3.06	299(5), **300**(100), 301(81), 302(8)	151(10), 179(8), 254(6), 255(54), 256(5), **271**(100), 272(15)	199(25), 203(11), 215(28), 227(68), 229(12), **243**(100)
**64**	Kaempferol 3-*O*-hexoside	5.31	C_21_H_19_O_11_^−^	447.09329	447.09067	2.61	255(15), 256(5), **284**(100), 285(57), 286(8), 316(7), 327(12)	227(14), **255**(100), 256(19), 257(4)	183(5), 187(5), 210(7), 211(62), 213(5), **227**(100)
**65**	Isorhamnetin 3-*O*-hexoside	5.37	C_22_H_21_O_12_^−^	477.10385	477.10104	2.81	271(6), 285(8), 300(6), **314**(100), 315(61), 316(7), 357(12)	243(24), 257(9), 271(77), **285**(100), 286(30), 299(41), 300(25)	**270**(100), 271(4)
**66**	Isorhamnetin 3-*O*-pentoside	5.64	C_21_H_19_O_11_^−^	447.09329	447.09051	2.78	271(4), 285(5), 286(3), **314**(100), 315(25), 316(4), 357(9)	243(30), 257(12), 271(77), **285**(100), 286(44), 299(12), 300(19)	**270**(100), 271(4)
**67**	Quercetin	6.36	C_15_H_9_O_7_^−^	301.03538	301.03305	2.32	107(6), 151(86), **179**(100), 180(8), 257(11), 271(32), 273(17)	**151**(100)	63(7), 65(3), 83(17), **107**(100)
**68**	Isorhamnetin	7.30	C_16_H_11_O_7_^−^	315.05103	315.04865	2.38	**300**(100), 301(9)	**151**(100), 227(40), 228(22), 255(31), 271(88), 272(66), 283(33)	63(3), 63(3), 65(6), 83(7), **107**(100)
***Anthocyanins and anthocyanidins***									
**69**	Cyanidin 3,5-di-*O*-hexoside 1	3.20	C_27_H_31_O_16_^+^	611.16066	611.16234	−1.67	287(97), 288(16), **449**(25), 449(100), 450(17)	**287**(100)	137(41), 175(27), 185(33), **213**(100), 231(45), 241(53)
**70**	Peonidin 3,5-di-*O*-hexoside	3.53	C_28_H_33_O_16_^+^	625.17631	625.17765	−1.34	301(70), 302(11), **463**(100), 464(14)	**301**(100)	**286**(100)
**71**	Cyanidin 3-*O*-hexoside 1	3.74	C_21_H_21_O_11_^+^	449.10784	449.10869	−0.85	**287**(100), 288(11)	137(41), 175(30), 185(30), **213**(100), 231(60), 241(52), 287(70)	129(7), 141(24), 143(9), 157(28), 167(11), 171(12), **185**(100)
**72**	Delphinidin 3,5-di-*O*-hexoside	3.74	C_27_H_31_O_17_^+^	627.15610	627.16114	−5.04	301(4), 303(22), **463**(7), 464(22), 465(100)	**303**(100)	137(24), 153(21), 165(55), 229(80), 247(26), **257**(100), 285(52)
**73**	Delphinidin 3-*O*-hexoside	3.74	C_21_H_21_O_12_^+^	465.10275	465.10378	−1.03	**303**(100)	137(24), 153(21), 165(55), 229(80), 247(26), **257**(100), 285(52)	NA
**74**	Delphinidin pentoside-hexoside	3.79	C_26_H_29_O_16_^+^	597.14501	597.14737	−2.35	301(13), 303(40), 435(15), 463(16), 464(21), **465**(100), 466(14)	**303**(100)	137(23), 165(55), 229(80), 247(29), **257**(100), 274(20), 285(51)
**75**	Cyanidin 3,5-di-*O*-hexoside 2	4.05	C_27_H_31_O_16_^+^	611.16066	611.16241	−1.75	287(27), **449**(26), 449(100), 450(7)	**287**(100)	121(42), 153(67), 165(95), 213(94), 231(35), **241**(100), 258(54)
**76**	Petunidin 3,5-di-*O*-hexoside	4.15	C_28_H_33_O_17_^+^	641.17123	641.17222	−0.99	317(21), 478(18), **479**(100), 480(8)	**317**(100)	139(8), 165(6), 257(10), 261(6), 274(8), 285(31), **302**(100)
**77**	Peonidin 3-*O*-hexoside	4.15	C_22_H_23_O_11_^+^	463.12349	463.12466	−1.18	**301**(100), 302(12)	258(4), **286**(100), 287(11)	202(10), 213(5), 229(10), 230(26), 257(22), **258**(100), 268(25)
**78**	Delphinidin rhamnoside-hexoside	4.51	C_27_H_31_O_16_^+^	611.16066	611.16296	−2.30	303(22), **449**(100)	**303**(100)	137(24), 153(22), 165(57), 229(89), 247(28), **257**(100), 285(54)
**79**	Petunidin	4.70	C_16_H_13_O_7_^+^	317.06558	317.06608	−0.50	**302**(100), 303(15)	228(58), 246(15), 256(22), 257(13), 273(32), 274(44), **285**(100)	187(8), 229(8), 239(20), **257**(100), 258(12), 267(17)
**80**	Cyanidin rhamnoside-hexoside	4.80	C_27_H_31_O_15_^+^	595.16575	595.16804	−2.29	287(30), 432(24), **433**(100), 434(4)	**287**(100)	121(37), 153(68), **165**(100), 213(94), 231(36), 241(97), 258(53)
**81**	Cyanidin 3-*O*-rhamnoside	4.85	C_21_H_21_O_10_^+^	433.11292	433.11405	−1.13	**287**(100), 288(63)	121(47), 153(77), **165**(100), 213(88), 241(90), 242(47), 258(57)	**109**(100), 123(57), 137(67)
**82**	Petunidin rhamnoside-hexoside	4.86	C_28_H_33_O_16_^+^	625.17631	625.17761	−1.30	317(20), **463**(100)	**317**(100)	139(8), 165(6), 257(9), 261(7), 274(7), 285(37), **302**(100)
**83**	Cyanidin rhamnoside-pentoside	4.90	C_26_H_29_O_14_^+^	565.15518	565.15669	−1.51	287(9), 419(5), **433**(100), 434(9)	**287**(100)	121(39), 153(73), **165**(100), 213(91), 231(35), 241(90), 258(49)
**84**	Delphinidin	4.96	C_15_H_11_O_7_^+^	303.04993	303.05051	−0.58	137(30), 165(67), 229(91), 230(31), **257**(100), 258(36), 285(51)	161(3), 173(4), 187(3), 201(10), 215(3), **229**(100), 230(7)	145(12), 159(5), 161(30), 173(30), 183(9), 187(19), **201**(100)
**85**	Cyanidin 3-*O*-hexoside 2	5.32	C_21_H_21_O_11_^+^	449.10784	449.10853	−0.70	**287**(100), 288(4)	121(37), 153(69), **165**(100), 213(92), 231(34), 241(96), 258(53)	69(18), **109**(100), 137(81)
**86**	Petunidin 3-*O*-hexoside	5.42	C_22_H_23_O_12_^+^	479.11840	479.11904	−0.64	**317**(100), 318(8)	139(9), 257(10), 261(7), 274(8), 285(37), **302**(100), 303(7)	153(29), 246(10), 273(16), **274**(100), 275(7), 284(15), 285(23)
***Terpene derivatives***									
**87**	Oxypaeoniflorin	3.61	C_23_H_27_O_12_^−^	495.15080	495.14670	4.10	245(18), 333(24), 447(92), 448(22), **465**(100), 466(22), 477(14)	137(31), 165(11), 179(12), 217(17), 281(27), **299**(100), 447(18)	89(68), 143(70), 206(68), **209**(100), 219(68), 226(68)
**88**	6′-*O*-Galloyl desbenzoyl paeoniflorin	3.72	C_23_H_27_O_14_^−^	527.14010	527.13640	3.70	345(19), **347**(100), 348(16), 365(18), 375(22), 479(13), 481(12)	125(25), 151(9), **169**(100), 195(10), 285(5), 303(3)	97(5), **125**(100)
**89**	Paeonin B	3.79	C_16_H_21_O_9_^−^	357.11911	357.11626	2.85	191(3), **195**(100), 196(9)	119(7), 123(15), 134(9), **135**(100), 136(89), 151(66), 177(20)	91(32), 91(41), **107**(100), 113(25)
**90**	Albiflorin + HCOOH	4.55	C_24_H_29_O_13_^−^	525.16137	525.15774	3.63	**449**(100), 479(34)	165(31), 309(7), **327**(100)	113(9), 123(10), **165**(100), 215(3), 309(22)
**91**	Galloyl paeoniflorin	5.11	C_30_H_31_O_15_^−^	631.16684	631.16273	4.12	271(21), 313(12), 479(13), 491(23), 509(8), **613**(100), 614(22)	211(23), 241(8), **271**(100), 313(38), 375(13), 399(17), 491(81)	169(11), **211**(100)
**92**	Paeoniflorin	5.76	C_23_H_27_O_11_^−^	479.15589	479.15289	2.99	151(4), 183(3), 195(13), 196(3), 213(7), **449**(100), 450(63)	137(4), 139(18), 140(6), 183(17), 184(4), **327**(100), 328(63)	**139**(100), 143(16), 163(24), 165(25), 183(74), 235(19), 237(21)
**93**	Benzoyl paeoniflorin + HCOOH 1	7.18	C_31_H_33_O_14_^−^	629.18758	629.18334	4.24	431(3), 552(6), **553**(100), 582(4), 583(67)	165(22), 265(6), 309(4), 413(8), 430(25), **431**(100), 525(4)	147(10), 162(9), **165**(100), 217(20), 243(8), 249(7)
**94**	Paeoniflorigenone	7.28	C_17_H_19_O_6_^+^	319.11762	319.11812	−0.51	161(3), 179(54), 180(4), **197**(100), 198(7), 300(4), 301(5)	123(7), 137(11), 151(6), 161(7), 167(16), **179**(100)	105(5), 123(8), **133**(100), 137(4), 149(12), 151(36), 161(41)
**95**	Benzoyl paeoniflorin + HCOOH 2	7.91	C_31_H_33_O_14_^−^	629.18758	629.18372	3.86	431(3), 535(4), 552(26), **553**(100), 554(4), 583(98), 584(3)	163(3), 165(30), 245(6), 291(3), 309(3), 413(9), **431**(100)	**165**(100), 171(29), 205(26), 217(45), 309(23)
***Other compounds***									
**96**	Citric acid	0.59	C_7_H_11_O_6_^−^	191.05560	191.04553	10.07	85(51), 93(28), **111**(100), 127(37), 129(13), 171(14), 173(36)	**67**(100), 81(32), 83(7), 93(7)	NA
**97**	Shikimic acid	0.73	C_7_H_9_O_5_^−^	173.04555	173.04418	1.36	93(86), **111**(100), 127(26), 128(11), 137(31), 143(28), 155(85)	64(4), 81(14), 83(46), **93**(100), 119(4), 126(4), 203(4)	NA
**98**	Apiopaeonoside	4.35	C_20_H_27_O_12_^−^	459.15080	459.14908	1.72	164(10), 269(9), **296**(100), 297(60), 310(21), 326(9), 327(17)	176(37), 180(35), 239(57), **240**(100), 251(35), 267(71), 268(69)	NA
**99**	Paeonoside	4.57	C_15_H_19_O_8_^−^	327.10854	327.10636	2.18	113(4), 123(12), 137(3), **165**(100), 166(6), 179(3), 309(15)	95(4), 121(12), 122(5), **123**(100), 137(8), 147(3), 150(5)	80(14), **81**(100), 95(30), 105(11), 108(25)
**100**	Paeonol	4.57	C_9_H_9_O_3_^−^	165.05572	165.05490	0.82	95(8), 121(7), 122(9), **123**(100), 137(11), 147(4), 150(4)	NA	NA
**101**	Picrocrocinic acid	5.22	C_16_H_27_O_8_^+^	347.17004	347.16996	0.09	107(6), 109(10), 125(8), 149(8), 167(21), 184(19), **185**(100)	107(12), 109(54), 125(62), 129(26), 149(19), 166(9), **167**(100)	**107**(100), 121(63), 123(57), 125(87), 137(45), 149(72), 245(56)
**102**	(+)-Paeonilactone B	6.40	C_10_H_13_O_4_^+^	197.08084	197.08109	−0.25	121(4), 133(12), 138(33), 139(3), **151**(100), 152(9), 179(8)	105(23), 123(5), **133**(100)	102(3), **105**(100)

NA—not available. The bold numbers indicate 100% of the base peak, as well as which peaks were further fragmented in the MS3 and MS4 experiments.

**Table 4:** Antibacterial activity of water and methanol extracts of the petals of *Paeonia peregrina* Mill. specimens with different origins (MIC and MBC, mg/mL).

Origin of Plant Material	Extraction Medium, Extraction Technique	Bacteria					
		*Staphylococcus lugdunensis*		*Proteus vulgaris*		*Staphylococcus aureus*	
		MIC	MBC	MIC	MBC	MIC	MBC
Pančevo	H_2_O, maceration	0.25	0.5	1	2	1	2
	MeOH, maceration	0.0625	0.125	1	2	1	2
	MeOH, UAE	0.125	0.25	0.25	0.5	0.25	0.5
Krivi vir	H_2_O, maceration	0.25	0.5	1	2	1	2
	H_2_O, UAE	0.25	0.5	1	2	1	2
	MeOH, maceration	0.25	0.5	1	2	1	2
	MeOH, UAE	0.25	0.5	0.5	1	0.5	1
	MeOH, MAE	0.25	0.5	0.5	1	0.5	1
Bogovo gumno	H_2_O, maceration	0.25	0.5	1	2	1	2
	H_2_O, UAE	0.25	0.5	1	2	1	2
	MeOH, maceration	0.125	0.25	0.5	1	0.5	1
Pirot	H_2_O, maceration	0.25	0.5	0.5	1	0.5	1
	H_2_O, UAE	1	2	1	2	1	2
	H_2_O, MAE	0.5	1	2	4	2	4
	MeOH, UAE	0.125	0.25	0.25	0.5	0.25	0.5
Gentamicin		0.008	0.016	0.066	0.133	1.33	2.66

Minimal inhibitory concentration (MIC); minimal bactericidal concentration (MBC); ultrasound-assisted extraction (UAE); microwave-assisted extraction (MAE); methyl alcohol (MeOH).

**Table 5:** Anticandidal activity of water and methanol extracts of the petals of *Paeonia peregrina* Mill. (MIC and MFC, mg/mL).

Origin of Plant Material	Extraction Medium, Extractiontechnique	*Candida* Species					
		*Candida kefyr*		*Candida krusei*		*Candida albicans*	
		MIC	MFC	MIC	MFC	MIC	MFC
Pančevo	H_2_O, maceration	0.5	1	1	2	0.5	1
	MeOH, maceration	0.25	0.5	1	2	0.125	0.25
	MeOH, UAE	0.5	1	0.5	1	0.5	1
Krivi vir	H_2_O, maceration	0.5	1	0.5	1	0.25	0.5
	H_2_O, UAE	0.5	1	1	2	0.5	1
	MeOH, maceration	1	2	0.5	1	0.5	1
	MeOH, UAE	0.5	1	1	2	0.5	1
	MeOH, MAE	0.5	1	1	2	0.125	0.25
Bogovo gumno	H_2_O, maceration	1	2	1	2	0.5	1
	H_2_O, UAE	1	2	1	2	0.5	1
	MeOH, maceration	0.5	1	1	2	0.25	0.5
Pirot	H_2_O, maceration	1	2	0.5	1	0.25	0.5
	H_2_O, UAE	1	2	1	2	1	2
	H_2_O, MAE	4	8	4	8	1	2
	MeOH, UAE	0.5	1	0.5	1	0.5	1
Ketoconazole		0.05	0.1	0.05	0.1	0.05	0.1

Ultrasound-assisted extraction (UAE); microwave-assisted extraction (MAE); methyl alcohol (MeOH); minimal inhibitory concentration (MIC); minimal fungicidal concentration (MFC).

**Table 6:** Effects of water and methanol extracts of the petals of *Paeonia peregrina* Mill. on *Staphylococcus lugdunensis* biofilms, expressed as a percentage of inhibition (%).

Origin of Plant Material	Extraction Medium, Extraction Technique	MIC	1/2 MIC	1/4 MIC
Pančevo	MeOH, maceration	NA	NA	14.28
	MeOH, UAE	NA	NA	NA
Bogovo gumno	MeOH, maceration	NA	NA	NA
Pirot	MeOH, UAE	NA	NA	NA

Minimal inhibitory concentration (MIC); ultrasound-assisted extraction (UAE); methyl alcohol (MeOH); no activity (NA).

**Table 7:** Effects of the water and methanol extracts of the petals of *Paeonia peregrina* Mill. on the denaturation of bovine serum albumin (BSA), expressed as the percentage of inhibition (%).

Origin of Plant Material	Extraction Medium, Extraction Method	Concentration, μg/mL	BSA Denaturation Inhibition, %
Pančevo	MeOH, maceration	1000	53.75 ± 0.46 ^ab^
		500	34.07 ± 0.74 ^b^
		250	18.64 ± 1.45 ^d^
	MeOH, MAE	1000	47.04 ± 2.28 ^ab^
		500	30.58 ± 3.00 ^c^
		250	17.30 ± 0,376 ^d^
Pirot	MeOH, MAE	1000	62.22 ± 0.77 ^a^
		500	43.42 ± 0.79 ^b^
		250	27.83 ± 0.16 ^c^
Control		100	20.84 ± 1.99 ^d^
		50	8.78 ± 0.54 ^e^
		25	6.28 ± 0.09 ^f^

Values with different letters (a–f) in each column showed statistically significant differences (*p* < 0.05; n = 3; analysis of variance, Duncan’s post hoc test); microwave-assisted extraction (MAE); methanol (MeOH).

## Data Availability

Data are contained within the article and Supplementary Materials.

## Supplementary Materials

The following supporting information can be downloaded at: https://www.mdpi.com/article/10.3390/ijms241411764/s1.
