# Supplementary material for: Paeonia peregrina Mill Petals as a New Source of Biologically Active Compounds: Chemical Characterization and Skin Regeneration Effects of the Extracts
Source: Int J Mol Sci. 2023 Jul 21;24(14):11764. doi: 10.3390/ijms241411764 (PMC10380736; doi:10.3390/ijms241411764)
Supplement: Supplementary file 1 [file ijms-24-11764-s001.zip › Supplementary Figure S1.pdf]

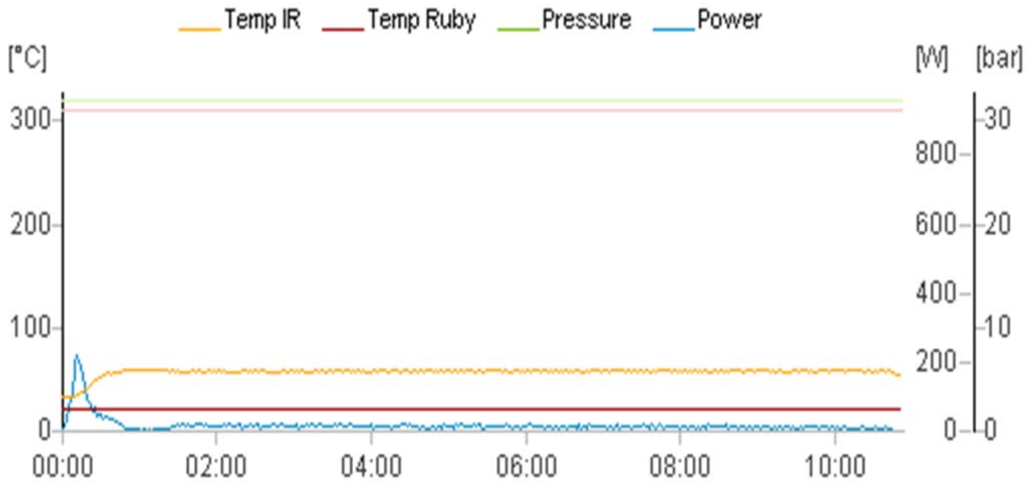

Supplementary Figure S1 Energy input (blue line – power [W]), pressure (green line – in bars) and temperature (yellow line – temp IR [°C])
