# Supplementary material for: Paeonia peregrina Mill Petals as a New Source of Biologically Active Compounds: Chemical Characterization and Skin Regeneration Effects of the Extracts
Source: Int J Mol Sci. 2023 Jul 21;24(14):11764. doi: 10.3390/ijms241411764 (PMC10380736; doi:10.3390/ijms241411764)
Supplement: Supplementary file 1 [file ijms-24-11764-s001.zip › Supplementary Figure S3.pdf]

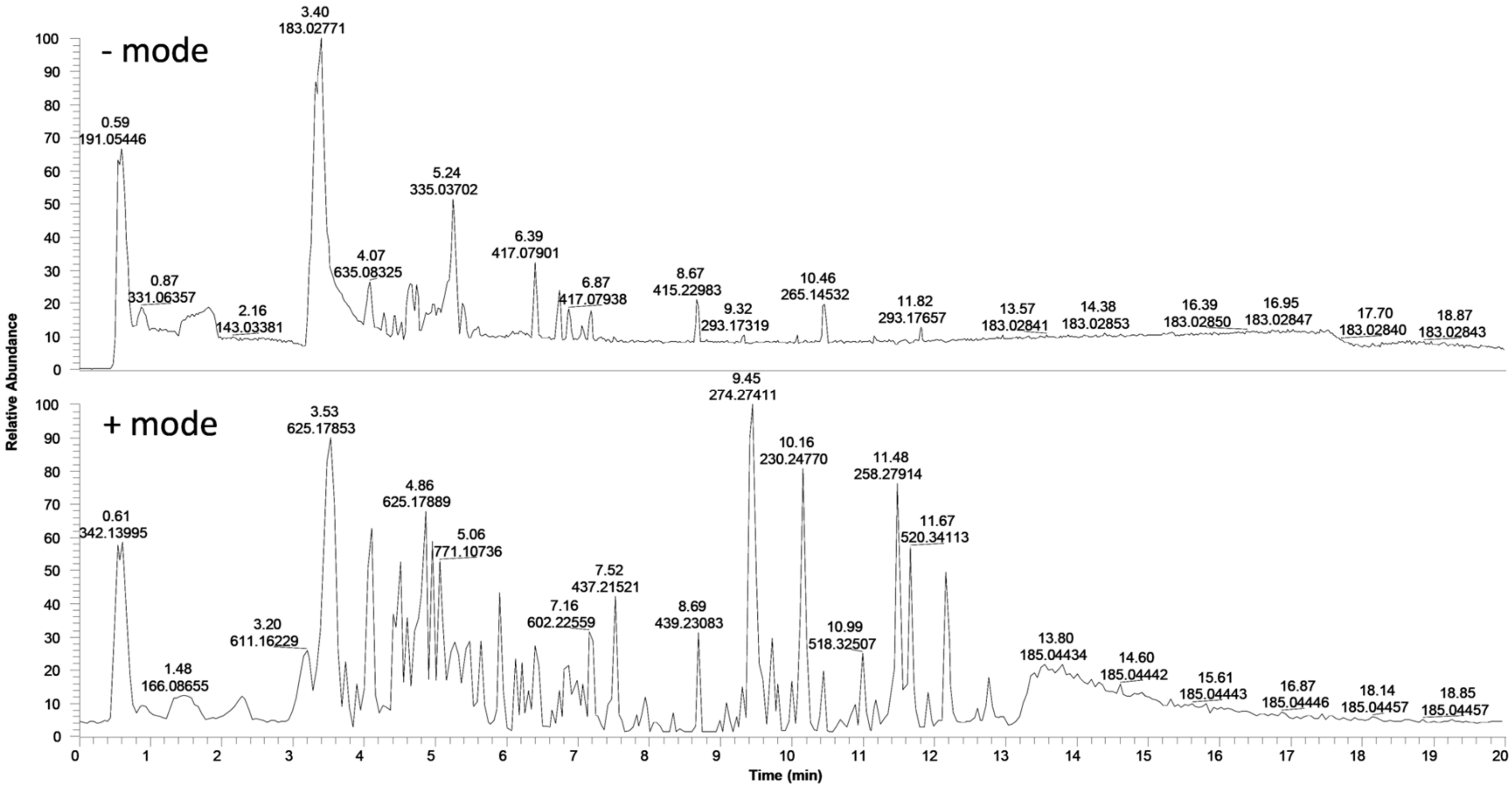

Supplementary Figure S3. Base peak chromatograms of *P. peregrina* in negative (top) and positive (bottom) ionization modes.
