# Supplementary material for: Paeonia peregrina Mill Petals as a New Source of Biologically Active Compounds: Chemical Characterization and Skin Regeneration Effects of the Extracts
Source: Int J Mol Sci. 2023 Jul 21;24(14):11764. doi: 10.3390/ijms241411764 (PMC10380736; doi:10.3390/ijms241411764)
Supplement: Supplementary file 1 [file ijms-24-11764-s001.zip › Supplementary Figure S5.pdf]

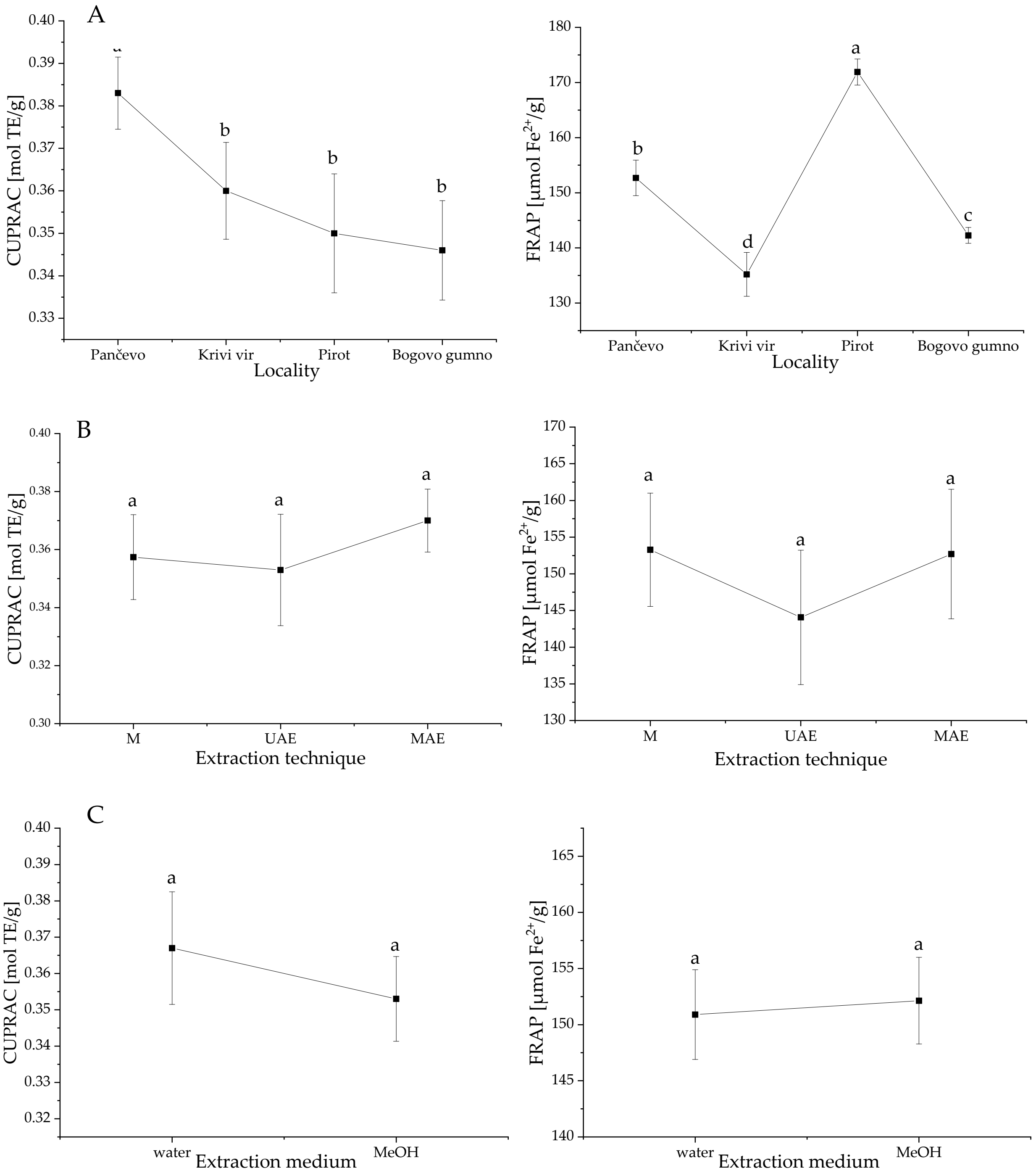

Supplementary Figure S5. Cupric ion reducing antioxidant capacity (CUPRAC) and ferric reducing antioxidant power (FRAP) of the extracts of *Paeonia peregrina* Mill. petals; the influence of A) localities (Pančevo, Krivi vir, Pirot, and Bogovo guvno), B) extraction techniques (maceration, M, ultrasound--assisted extraction, UAE, and microwave-assisted extraction MAE), and C) extraction mediums (water and methanol, MeOH); values with the same letter showed no statistically significant difference ($p > 0.05$; $n = 3$, one-way ANOVA, analysis of variance, Duncan's *post-hoc* test).
